# Major trauma and comorbidity: a scoping review

**DOI:** 10.1007/s00068-025-02805-x

**Published:** 2025-03-12

**Authors:** Rosie Glynn, Felicity Edwards, Martin Wullschleger, Ben Gardiner, Kevin B. Laupland

**Affiliations:** 1 Queensland University of Technology (QUT), Brisbane, QLD Australia; 2 Department of Intensive Care Services, Royal Brisbane and Women’s Hospital, Brisbane, QLD Australia; 3 Faculty of Medicine, University of Queensland, Herston, QLD Australia; 4 Trauma Services, Gold Coast University Hospital and School of Medicine, Gold Coast, Parkland, QLD Australia; 5 School of Medicine, Griffith University, Gold Coast, Brisbane, QLD Australia; 6 Healthcare Improvement Unit, Clinical Excellence Queensland, Queensland Health, Herston Queensland, Australia

**Keywords:** Global burden of disease, Case fatality rate, Wounds and injuries, Trauma, Physical, Comorbidity

## Abstract

**Purpose:**

Major trauma is a leading cause of acute morbidity and mortality. While injury severity drives much of the associated burden, pre-existing comorbidities may influence both acute management and long-term outcomes. This scoping review examines the impact of comorbidities on trauma outcomes.

**Methods:**

Embase, Medline, CINAHL, Cochrane Library, and PubMed were systematically searched from inception to 22/04/2021 (update 22/03/2024). Studies investigating comorbidities as risk factors for adverse outcomes in adults (≥ 18 years) with major trauma were included.

**Results:**

Of 5448 studies identified, 33 met inclusion criteria. No studies examined whether comorbidities increases the risk of major trauma, and only two studies investigated the development of comorbidities post-trauma. Among trauma patients with pre-existing comorbidities particularly cardiovascular disease, diabetes, liver disease, and kidney disease were associated with higher case fatality. Comorbidities were also associated with increased morbidity, longer hospital stays and higher complication rates.

**Conclusions:**

Trauma patients with comorbidities suffer experience worse outcomes, yet limited research explores whether comorbidities contribute to trauma risk or emerge as a consequence. Further research is needed to clarify these relationships and guide targeted interventions.

**Supplementary Information:**

The online version contains supplementary material available at 10.1007/s00068-025-02805-x.

## Introduction

Major trauma is a leading cause of morbidity and mortality worldwide [1]. While younger individuals experience the highest incidence and disability adjusted life years due to trauma [2], older adults also represent a significant and growing demographic affected by major trauma [3]. With global populations aging, the burden of trauma in older individuals is expected to rise substantially [4, 5].

In contrast to children and young adults, older adults frequently have pre-injury comorbidities that can complicate trauma management, prolong hospital stays, and contribute to adverse outcomes [6, 7]. These pre-injury comorbidities and their treatments may lead to functional impairments that increase the risk for experiencing traumatic injuries [8, 9]. Further, trauma related injuries, particularly those involving organ damage, may predispose individuals to post trauma chronic medical conditions such as cardiovascular disease following severe chest trauma or chronic kidney disease after an acute kidney injury [10].

Despite the potential for comorbidities to influence trauma risk and outcomes, and the possibility of trauma related injuries leading to post trauma comorbidities, there is limited literature examining these interrelationships [11]. Previous systematic reviews have explored trauma and comorbidities [12, 13], but these have been limited to specific comorbidities or selected types of injuries. To our knowledge no reviews have specifically examined major trauma as a risk factor for the development of post trauma comorbidities. Therefore, this study aimed to conduct a scoping review to explore the impact of comorbidities on trauma burden and outcomes to identify priorities for future research.

### Review questions

This review sought to answer the following research questions:

1. Do pre-injury comorbidities increase the risk of experiencing major trauma?
2. Do pre-injury comorbidities increase the morbidity associated with major trauma?
3. Do pre-injury comorbidities increase case fatality associated with major trauma?
4. Does major trauma increase the risk of developing post trauma comorbidities in the long-term?

### Methods

This scoping review followed the methodological framework established by the Joanna Briggs Institute and its Collaborating Centers [14]. The protocol was developed *a priori.*

Studies were eligible for inclusion if they investigated one or more of the research questions, involved patients aged ≥ 18 years, examined major trauma cases, and assessed the association between trauma and at least one comorbidity. Additionally, studies that met all inclusion criteria but did not directly address a pre-specified research question were also considered. This approach was taken to allow concepts and themes that emerged and had not been considered a priori to be included.

Studies focusing solely on isolated extremity injuries, chronic pain, functional outcomes, quality of life, substance use disorders, and/or psychiatric conditions including post-traumatic stress disorder (PTSD) were excluded. Further case reports, editorials, review articles, and reports that were published only as abstracts were excluded. No language restriction was applied on the initial search however, articles without readily accessible translation were excluded.

Major trauma was defined as an acute traumatic injury meeting severity criteria such as an injury severity score (ISS) ≥ 12 or requiring admission to an intensive care unit (ICU) [15]. Pre-injury comorbidities were defined as medical conditions already present before the trauma event, as categorised using validated scoring systems such as the Charlson Comorbidity Index (CCI), or the Acute Physiology and Chronic Health Evaluation (APACHE) system, or as explicitly stated in the included studies [16, 17].

For the purposes of this review, morbidity was defined as any post trauma complication or adverse health outcome that prolonged hospitalisation, required additional medical intervention, or resulted in long term functional impairment [18]. Case fatality was defined as mortality occurring either during the initial hospitalisation or within a defined follow-up period due to complications arising from the traumatic event [18].

An initial electronic search was conducted in Embase (Elsevier) by a librarian using Medical Subject Headings (MeSH) and keywords related to major trauma and comorbidities. This search was reviewed using the PRESS Checklist and subsequently translated into searches in Medline (EBSCOhost), CINAHL (EBSCOhost), Cochrane Library and PubMed [14]. The initial search was conducted on April 22nd 2021, and results were deduplicated in EndNote X9.2 (Clarivate Analytics, PA, USA). Additional studies were identified by screening the reference list of included articles, review articles, and editorials. The search was updated on March 22nd 2024 to include newly published literature.

Titles and abstracts were screened independently by two authors (RG and FE) using Colandr [19]. Studies meeting inclusion criteria underwent full text review by the same reviewers. Discrepancies were resolved by consensus. The variables extracted included author name, year of publication, study setting, country of origin, study population and sample size, study design, research question addressed as shown in Table 1.

**Table 1:** Characteristics of included studies

Author and Year of Publication	Country of origin	Study Objective	Study Design	Study Population	Key Findings
Bala et al. 2013	Israel	To identify pre-hospital and admission parameters that predict in-hospital mortality among trauma patients aged ≥ 60 years.	Retrospective review.	417 trauma patients aged ≥ 60 years admitted between January 2006 and December 2010.	Among severely injured trauma patients, pre-existing comorbidities significantly influenced in-hospital mortality. Chronic renal failure was associated with a higher case fatality rate.
Bergeron et al. 2005	Canada	To determine the separate and combined effects of age and comorbidities on the length of hospital stay for trauma patients in a tertiary trauma center.	Retrospective review.	994 trauma patients, aged > 14 years, survived hospital discharge between April 1, 2000, and March 31, 2001.	Trauma patients with comorbidities had nearly double the hospital stay compared to those without. Age comorbidity presence and injury severity were independent predictors of length of stay with neurologic and pulmonary conditions contributing most.
Blair et al. 2022	Cameroon	To assess the prevalence of comorbidities among injured patients and evaluate their association within hospital outcomes, including complications and mortality.	Retrospective cohort study utilising data from a prospective trauma registry.	The study analysed data from 7,509 patients recorded in the trauma registry between October 2017 and January 2020.	Patients with comorbidities had higher rates of complications with wound infections, urinary tract infections and decubitus ulcers the most common Comorbidities were independently associated with increased odds of mortality. Among patients with severe injuries (ISS 16–24) those with comorbidities had higher case fatality.
Brennan et al. 2002	Australia	To identify factors predictive of case fatality among trauma patients and assess trends in case fatality over the first four years of operation of South Australia’s major trauma system.	Retrospective cohort study analysing trauma registry data.	The study included 8,654 trauma patients admitted to major trauma services in South Australia between 1997 to 2000.	Older age, higher ISS and the presence of comorbidities (Chronic liver disease, ischemic heart disease and chronic renal failure) were significant predictors of case fatality.
Childs et al. 2015	United States	To examine the impact of obesity on complication rates and hospital LOS in patients with trauma.	Retrospective cohort study.	The study included 376 patients treated for injuries at a Level 1 Trauma center over a 30-month period.	Obese patients had longer LOS and required more time on mechanical ventilation compared to non-obese patients. Complication rates were significantly higher in the obese group. The study concluded that obesity is associated with increased morbidity and prolonged hospitalisation following trauma.
de Vries et al. 2019	Netherlands	To describe the mortality pattern of older polytrauma patients, identify associated risk factors, and examine the role and etiology of in-hospital complications.	Retrospective cohort study.	The study included 385 polytrauma patients aged > 65 years admitted between 2008 and 2016.	For patients with polytrauma, comorbidities did not significantly affect mortality.
Duvall et al. 2015	United States	To determine whether injury severity and pre-existing comorbidities can predict the futility of care in elderly trauma patients.	Retrospective cohort study analysing trauma database.	The study included 570,442 patients aged > 70 years between 2007 and 2011.	The study found that neither ISS nor the presence of pre-existing comorbidities alone could predict futility of care, defined as an in-hospital mortality rate of 95% or higher, in severely injured elderly trauma patients.
Elkbuli et al. 2019	United States	Assess the impact of comorbidities on mortality and LOS on trauma patients.	Retrospective Study.	The study included 9,845 patients admitted to a trauma centre between January 2014 and December 2016.	In the ISS ≤ 15 group, patients with fewer than three comorbidities had a significantly higher mortality rate compared to those with 1–2 comorbidities. Comparing patients with ISS ≤ 15 and ≥ 3 comorbidities to those with ISS > 15 and 1–2 comorbidities, the mortality rate was significantly higher in the former group. The ICU LOS was significantly longer in the ISS ≤ 15 group with ≥ 3 comorbidities compared to those with 1–2 comorbidities. The study concluded that increased comorbidities are associated with significantly higher mortality and longer ICU stays, suggesting that comorbidities may serve as markers of lower physiological reserve and should be considered in trauma scoring systems to improve outcome predictions.
Gabbe et al. 2005	Australia	To evaluate the effectiveness of the CCI in predicting mortality outcomes in trauma patients.	Retrospective cohort study.	The study population consisted of major trauma patients (*n* = 2,819) from a data registry.	The researchers found that while the CCI was associated with mortality, adding the CCI to existing trauma prediction models did not significantly improve their performance. Therefore, they concluded that the CCI alone may not be a sufficient predictor of trauma outcomes.
Gelaw et al. 2022	Australia	To determine the prevalence of chronic physical health conditions reported pre-injury, at the time of injury, up to 1-year post-injury, and 1 to 5 years post-injury; and to assess the risk of these conditions in individuals with orthopedic major trauma compared to other types of major trauma.	Retrospective cohort study utilising registry data.	The study included 28,522 major trauma patients aged 18 years and older, who were registered by the Victorian State Trauma Registry between 2007 and 2016 and survived at least one-year post-injury.	The prevalence of comorbidities among patients was 69.3%. The most common were arthritis and arthropathies, cancer, and cardiovascular diseases. The highest prevalence of new-onset conditions after injury was observed in individuals with severe traumatic brain injury and orthopedic major trauma, while the lowest was in those with other types of major trauma. There were no significant differences in the adjusted risk of conditions reported 1 to 5 years post-injury between the orthopedic injury group and other major trauma groups. The study concluded that chronic physical health conditions are common among all injury groups, and rehabilitation practitioners should be aware of this risk. Long-term follow-up care after injury should include prevention and treatment of chronic conditions.
Georgiou et al. 2009	United States	To assess the impact of pre-existing cirrhosis on trauma patient outcomes, particularly focusing on mortality and complication rates.	Retrospective cohort study utilising registry data.	The study included 468 trauma patients diagnosed with cirrhosis, admitted between 1997 and 2006.	Cirrhotic trauma patients had significantly higher case fatality rates than non-cirrhotic patients. ICU LOS was longer in cirrhotic patients and they had a significantly higher rate of complications. The study concluded that cirrhosis is a major risk factor for poor trauma outcomes, necessitating specialised management strategies for this high-risk population.
Hollis et al. 2006	United Kingdom	To investigate the impact of pre-existing comorbidities and age on mortality following traumatic injury, and to explore how these factors interact with injury severity.	Retrospective analysis of trauma registry data.	A total of 65,743 trauma patients were admitted to hospitals within the trauma network.	The study found that the presence of comorbidities significantly increased mortality in patients with moderate injuries but had a less pronounced effect in those with severe injuries.
McGwin et al. 2004	United States	To develop and validate a predictive model for mortality in burn patients by incorporating variables such as age, percentage of total body surface area burned (TBSA), inhalation injury, co-existent trauma, and pneumonia.	Retrospective cohort study utilising registry data.	The study analysed data from 68,661 burn patients between 1994 and April 2002.	The predictive model that included age, TBSA, inhalation injury, co-existent trauma, and pneumonia demonstrated optimal performance. The inclusion of additional variables, such as gender and comorbidities, did not significantly improve the model’s performance. The study concluded that incorporating specific variables not previously considered in other models provides superior predictive ability for burn mortality
Milzman et al. 1992	United States	To determine whether pre-existing comorbidities are independent predictors of mortality in trauma patients, beyond the influences of age and ISS.	Retrospective cohort study.	The study analysed data from 7,798 adult trauma patients admitted between July 1986 and June 1990.	Patients with pre-existing comorbidities were older and had a higher mortality rate compared to those without. Specific conditions such as renal disease, malignancy, and cardiac disease were associated with notably higher mortality rates. After adjusting for age and ISS, the presence of pre-existing comorbidities remained an independent predictor of mortality. The study concluded that pre-existing comorbidities significantly impact trauma outcomes, suggesting a need for tailored management strategies for these patients.
Mira et al. 2021	United States	To determine the incidence and risk factors associated with the development of chronic critical illness (CCI) in patients who have suffered severe blunt traumatic injuries.	Prospective, observational study.	The study included 135 adult patients who experienced severe blunt trauma accompanied by hemorrhagic shock and survived beyond 48 h post-injury between 2013 and 2016.	Approximately 19% of the patients developed CCI, defined as an ICU stay of 14 days or more with ongoing organ dysfunction. Factors independently associated with the progression to CCI included age 55 years or older, severe shock the need for transfusion of 5 or more units of packed red blood cells within the first 24 h, and higher organ failure severity scores at 72 h post-injury. Patients who developed CCI had higher rates of infectious complications, increased in-hospital mortality, and were more frequently discharged to long-term care facilities compared to those who recovered more rapidly. The study concluded that CCI is a common outcome in survivors of severe trauma and is associated with poor long-term outcomes, highlighting the importance of early identification and targeted interventions for at-risk patients.
Moore et al. 2008	Canada	To assess whether incorporating information on preexisting comorbidities improves the accuracy of mortality predictions in trauma patients.	Retrospective cohort study.	The study included trauma patients from a regional trauma registry; (*n* = 22,744), however, dates of study period unknown.	The study found that including preexisting comorbidities in predictive models enhanced the accuracy of mortality predictions in trauma patients. The authors concluded that accounting for preexisting comorbidities is important for improving the precision of mortality risk assessments following traumatic injury.
Niven et al. 2012	Canada	To assess the impact of pre-existing comorbidities on one-year mortality in adult patients who have suffered major traumatic injuries.	Retrospective population-based cohort study.	The study included 3,080 adult trauma patients admitted between April 1, 2002, and March 31, 2006. The median Injury Severity Score was 20.	After adjusting for age, the CCI was independently associated with increased one-year mortality, with an OR of 1.24 per point on the CCI. A predictive model incorporating both age and CCI accurately predicted one-year mortality. The study concluded that pre-existing comorbidities significantly influence long-term outcomes after major trauma, suggesting a need for enhanced medical co-management in this patient population.
Passman et al. 2020	United States	To investigate whether fragmentation of care, defined as readmission to a different hospital from the index admission, is associated with increased mortality among trauma patients.	Retrospective cohort study.	The study analysed data from patients who were readmitted within 30 days of discharge between 2011 and 2015. Patients were categorised based on whether they were readmitted to the same hospital (non-fragmented care) or a different hospital (fragmented care).	Patients with comorbidities at 30-day readmission had higher mortality rates than those with no comorbidities.
Peng et al. 2015	United States	To assess the mortality rate among patients with an ISS of 75 and examine their characteristics and primary diagnoses.	Retrospective cohort study of registry data.	The study analysed 2,815 patients with an ISS of 75, between 2006–2010 representing an estimated 13,569 patients nationwide.	Non-survivors were more likely to have at least one comorbidity.
Pratt et al. 2021	Canada	To assess the impact of chronic kidney disease (CKD) and dialysis on in-hospital mortality among major trauma patients.	Retrospective cohort study of registry data.	The study included 6,237 major trauma patients admitted between 2006 and 2017. Of these, 101 had stage 3–5 CKD or were receiving dialysis, while 4,896 did not have CKD.	Patients with CKD or on dialysis had a higher in-hospital mortality rate compared to those without CKD. After adjusting for age, sex, and injury severity, CKD/dialysis patients had nearly double the risk of in-hospital case fatality. The study concluded that CKD and dialysis are independent risk factors for increased in-hospital mortality following major trauma.
Selassie et al. 2013	United States	To evaluate the influence of comorbid diseases and concomitant injuries on the risk of in-hospital death after traumatic spinal cord injury (SCI).	Retrospective population-based cohort study.	A total of 3,389 patients were admitted between January 1998 and December 2009.	The study found that certain factors significantly increased the risk of in-hospital mortality following acute traumatic SCI. These factors included the presence of comorbidities. The findings suggest that both pre-existing comorbidities and the nature of the injury itself play critical roles in patient outcomes after SCI. These results underscore the importance of comprehensive clinical assessments that consider both comorbidities and injury characteristics to improve management strategies and reduce in-hospital mortality rates among SCI patients.
Shoko et al. 2010	Japan	To assess the impact of pre-existing comorbidities on in-hospital mortality among trauma patients in Japan.	Retrospective cohort study of registry data.	A total of 20,257 trauma patients aged 16 years and older, admitted to hospitals in Japan between 2004–2007.	Specific comorbidities including cirrhosis, active cancer, chronic obstructive pulmonary disease, hematologic disorders, use of anticoagulation drugs, dementia or mental retardation, were associated with higher in-hospital mortality. Having two or more comorbidities significantly increased mortality risk, particularly in patients aged 50 to 74 and those with minor injuries.
Shu et al. 2022	Australia	To assess the impact of pre-existing comorbidities on survival outcomes following major injuries among different types of road users.	Retrospective cohort study analysing registry data.	The study population included 6,253 patients who sustained major injuries from road traffic incidents between 2013–2019.	Key findings indicated that the presence of comorbidities adversely affected survival rates post-injury across all road user types. The study highlighted the importance of considering pre-existing comorbidities in trauma care and the need for tailored management strategies to improve outcomes for injured patients with comorbidities.
Stewart et al. 2020	United States	To determine the impact of traumatic injury on the subsequent development of hypertension, diabetes mellitus, and coronary artery disease after adjusting for sociodemographic, health behavior, and mental health factors.	Retrospective cohort study analysing registry data.	The study included 8,727 combat-injured service members between February 2002, and June 2016, matched 1:1 based on year of birth, sex, and branch of service to individuals who deployed to a combat zone but were not injured.	After adjustments, severe traumatic injury was significantly associated with increased risks of hypertension, diabetes and coronary artery disease compared to no injury. Less severe injuries were also associated with higher risks of hypertension and coronary artery disease. These findings suggest that severe traumatic injury is linked to the subsequent development of comorbidities, highlighting the need for targeted primary care interventions for injured service members.
Su et al. 2019	Taiwan	To evaluate the impact of admission hyperglycemia on mortality rates among adult patients with moderate-to-severe thoracoabdominal injuries.	Retrospective cohort study analysing registry data.	The study included 752 patients admitted to a level 1 trauma center between January 2009 and December 2018.	The study found that both stress induced hyperglycemia and diabetic hyperglycemia patients had significantly higher mortality rates compared to nondiabetic normoglycemia patients, even after adjusting for age, sex, comorbidities, and injury severity. However, there was no significant difference in mortality rates between the groups. These findings suggest that admission hyperglycemia, regardless of its etiology, is associated with increased mortality in patients with thoracoabdominal injuries.
Taylor et al. 2002	United States	To assess intensive care unit (ICU) resource utilisation and outcomes in elderly trauma patients.	Retrospective cohort study analysing registry data.	The study population included 26,237 elderly patients admitted between Jan 1996 - Dec 1997.	Patients with pre-existing comorbidities (cardiovascular disease, diabetes, cancer, respiratory disease) were at a significantly higher risk of mortality.
Toomey et al. 2014	United States	To assess the impact of cancer presence, specific cancer types, and metastasis on in-hospital mortality rates among patients aged 50 and older who sustained fall-related injuries.	Retrospective cohort study analysing registry data.	The study included 4,201 cancer patients and an equal number of non-cancer patients, all aged between 50 and 96 years, who were hospitalised due to fall-related injuries.	After adjusting for various factors, cancer patients exhibited a higher likelihood of in-hospital mortality following a fall compared to non-cancer patients. Patients with metastatic cancer faced an even greater risk of death while those without metastasis also had an elevated risk. Notably, individuals with cancers at sites other than the prostate and breast demonstrated significantly higher mortality rates. The study concluded that cancer patients, particularly those with metastasis or certain cancer types, are at an increased risk of in-hospital death after experiencing a fall.
Varachhia et al. 2020	Trinidad and Tobago	To identify factors influencing mortality among major trauma patients in Trinidad and Tobago.	Retrospective cohort study analysing registry data.	The study included 323 patients admitted between 2010 and 2014.	The study found that multiple factors influence mortality including age, co-morbidities, and injury mechanism.
Wang et al. 2018	Taiwan	To evaluate the impact of comorbidities on the prognosis of trauma patients.	Retrospective cohort study analysing registry data.	The study included 4,997 patients with trauma admitted to a Level I trauma center between January 2011 and December 2015.	The researchers utilised the Index of Coexistent Comorbidity Disease (ICED) scores to assess comorbidity severity. They found that patients with higher ICED scores had a significantly higher in-hospital mortality rate compared to those with lower scores. This association was particularly evident in patients with an ISS below 25, suggesting that comorbidity severity plays a crucial role in outcomes, even among those with less severe injuries.
Wong et al. 2018	Singapore	To develop a predictive model for 1-year and 3-year mortality among adult survivors of major blunt trauma.	Retrospective cohort study analysing registry data.	Patients who sustained blunt injuries with an Injury Severity Score (ISS) of 12 or more including 3,414 patients admitted between 2011–2013	Key findings indicated that factors such as advanced age, male gender, low fall mechanisms (from 0.5 m or less), higher Charlson Comorbidity Index scores, presence of diabetes, cancer, severe head and neck injuries (AIS ≥ 3), and prolonged hospital stays (≥ 30 days) were significant predictors of increased mortality at both 1-year and 3-year intervals.
Wutzler et al. 2009	Germany	To assess the impact of preexisting medical conditions on in-hospital mortality among patients with multiple traumatic injuries.	Retrospective cohort study.	The study included 11,142 patients between 2002–2007.	The study found that pre-existing comorbidities are associated with increased in-hospital mortality in multiple-trauma patients. This suggests that pre-existing comorbidities should be considered when evaluating patient prognosis and treatment strategies.
Yiannoullou et al. 2017	United Kingdom	To assess the impact of regional trauma networks on the management and outcomes of blunt splenic injuries	Retrospective cohort study analysing registry data.	The study analysed 1,572 patients between April 2010 to March 2014.	Key findings include a significant increase in the use of splenic artery embolisation and a decrease in splenectomy rates after the establishment of trauma networks. Despite these changes, overall mortality rates remained similar between the two periods. The study suggests that the implementation of regional trauma networks has led to increased splenic preservation through greater use of embolisation techniques.

*Abbreviations* ISS – Injury severity score, OR – Odds ratio, LOS – Length of stay, ICU – intensive care unit, CCI – Charlson Comorbidity Index

Analysis was descriptive and all reported study statistics were taken at face value without further validation. When not provided in the original studies, relative risks (RR) were calculated using available data on event rates in exposed and unexposed groups. Specifically, RR was computed as the ratio of the probability of the outcome occurring in individuals with the given exposure (e.g. pre-injury comorbidity) compared to those without that exposure.

To improve comparability, RR values were grouped with hazard ratios (HR) and odds ratios (OR), acknowledging that while these measures differ in interpretation, they provide insights into risk associations. Odds ratios from included studies were also synthesised into forest plot to visually represent the association between comorbidities and trauma outcomes. Studies were stratified based on whether they examined the impact of a single comorbidity or two or more comorbidities. Results were organised by research questions to provide structured synthesis of findings. Additionally, studies were categorised based on whether they examined pre-injury comorbidities, trauma related injuries or post trauma comorbidities to ensure consistency in interpretation.

## Results

**Fig. 1:**
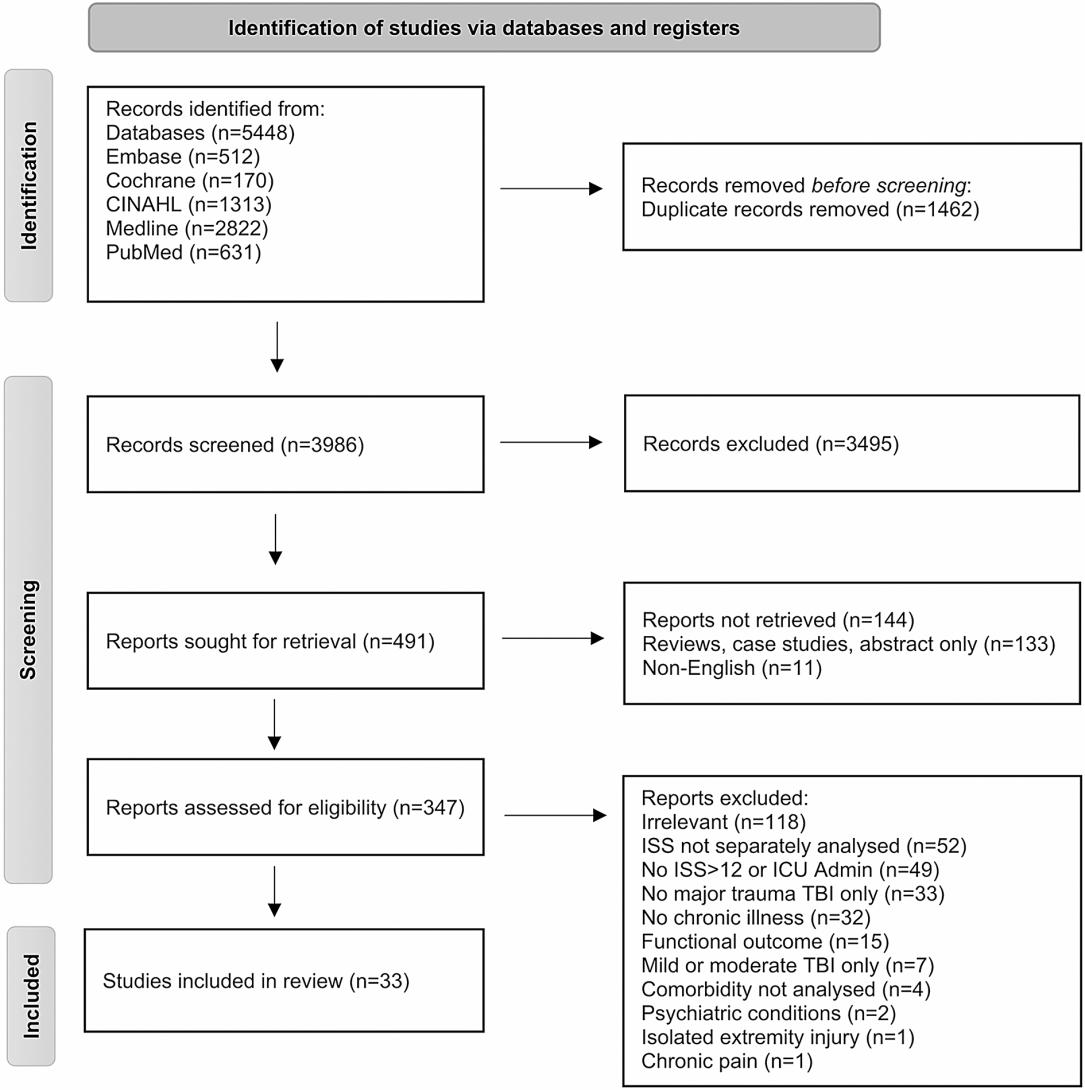
Search results and study selection and inclusion process [14]

The electronic search retrieved 5,448 citations from PubMed (631), Medline (2,822), CINAHL (1,313), Cochrane (170) and Embase (512) databases. After removing duplicates and applying inclusion and exclusion criteria, 33 articles were included in the final review, as detailed in Fig. 1. An additional relevant study was included after repeating the search on March 22nd, 2024. The years of publication ranged from 1992 to 2023.

The studies were conducted across 12 countries, most frequently from the United States of America (*n* = 13; 39.4%), followed by Canada (*n* = 4; 12.1%), Australia (*n* = 4; 12.1%), Taiwan (*n* = 2; 6.1%), United Kingdom (*n* = 2; 6.1%), and one paper published each from Cameroon, Turkey, Netherlands, Israel, Japan, Germany, Singapore, and Trinidad and Tobago (*n* = 1; 3.0%). Two studies prospectively recruited patients whilst the remaining studies used retrospective data obtained from trauma registries, or electronic medical records. Review findings are organised according to the predefined research question.

### Question 1: do pre-injury comorbidities increase the risk of experiencing major trauma?

No identified studies examined pre-injury comorbidities as risk factors for development of major trauma.

### Question 2: do pre-injury comorbidities increase the morbidity associated with major trauma?

Six studies investigated the relationship between pre-injury comorbidities and length of stay (LOS) in trauma patients [20–25]. A prospective observational cohort study of 135 patients found that those with pre-injury comorbidities had significantly longer ICU LOS compared to those without (27 vs. 8 days; *p* < 0.0001) [20]. Similarly, a retrospective review of 9,845 trauma patients with a ISS > 15 found no significant difference in LOS for patients with three or more pre-injury comorbidities compared to those with one or two (31 vs. 29 days; *p* > 0.0500) [22].

A study of 994 trauma patients found that those with a CCI of one had a longer hospital stay than those without a pre-injury comorbidities (29 vs. 15 days; *p* < 0.0010) [23]. Additionally, this study reported that patients with pulmonary comorbidities had a significantly longer adjusted mean LOS compared to those without (28 vs. 19 days; *p* = 0.0100) [23]. Similarly Georgiou et al., found that trauma related injuries in patients with cirrhosis resulted in longer ICU LOS (5 vs. 8 days; *p* < 0.0001) and higher complication rates (10% vs. 4%; *p* < 0.0001) compared to non-cirrhotic patients [24]. Another study by Pratt et al. analysing 6,237 trauma patients found that pre-injury chronic kidney disease and/or dialysis did not significantly impact hospital LOS (14 vs. 11 days; *p* = 0.1200) or duration of mechanical ventilation (5 vs. 4 days; *p* = 0.0900) [25]. A cohort study of 376 trauma patients reported those with obesity had longer LOS (12 vs. 10 days; *p* < 0.0100) and longer duration on mechanical ventilation (5 vs. 3 days; *p* < 0.0100) compared to non-obese patients [21].

Two additional studies found pre-injury comorbidities were associated with higher complication rates. Mira et al.. reported that trauma patients with two or more pre-injury comorbidities developed a chronic critical illness compared to those without comorbidities (44% vs. 28%; *p* < 0.0500) [20]. Similarly, Blair et al.. found that patients with pre-injury comorbidities had more complications (19% vs. 14%; *p* = 0.1920) with wound infections, urinary tract infections and decubitus ulcers being the most common [26].

### Question 3: do pre-injury comorbidities increase case fatality associated with major trauma?

Most studies in this review examined the association between pre-injury comorbidities and case fatality following major trauma. A large proportion evaluated the overall impact of comorbidities on trauma related case fatality [26–43], while another subset of studies investigated the effect of specific comorbidities [24, 25, 27, 28, 31–33, 37, 39, 40, 44–48].

### Overall comorbidities

**Fig. 2:**
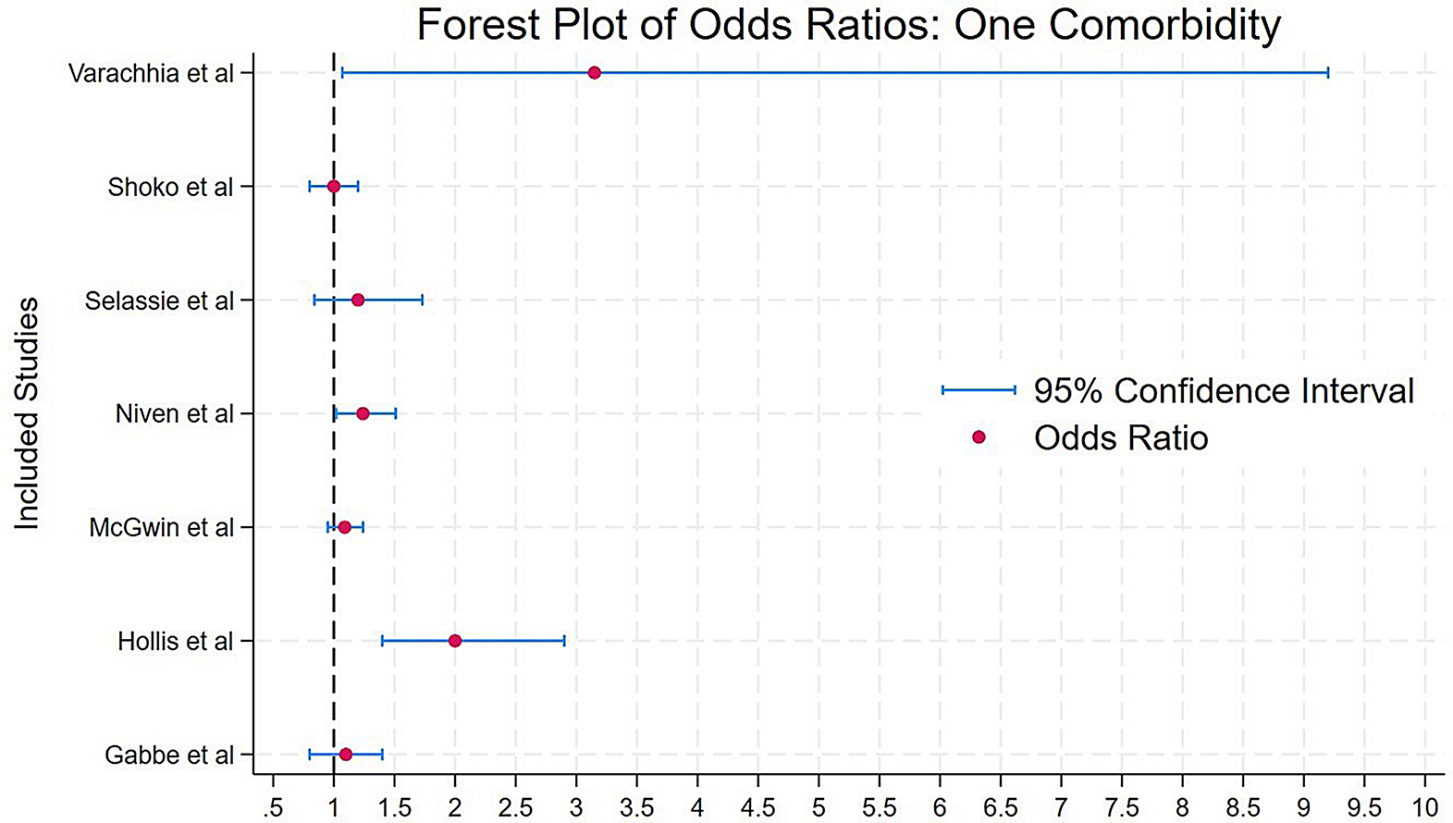
Forest plot of odds ratios (with 95% confidence intervals) for mortality in patients with one comorbidity, as reported in various studies **Shortlist of Explanations**: This forest plot displays odds ratios (OR) and 95% confidence intervals (CI) from studies examining mortality risk in trauma patients with one comorbidity. The vertical dashed line at OR = 1 represents the reference value (no effect). OR values to the right of this line (> 1) indicate an increased mortality risk, while values to the left (< 1) would suggest a decreased risk. Most studies show OR values above 1, suggesting that having a single comorbidity is associated with higher mortality risk in trauma patients

**Fig. 3:**
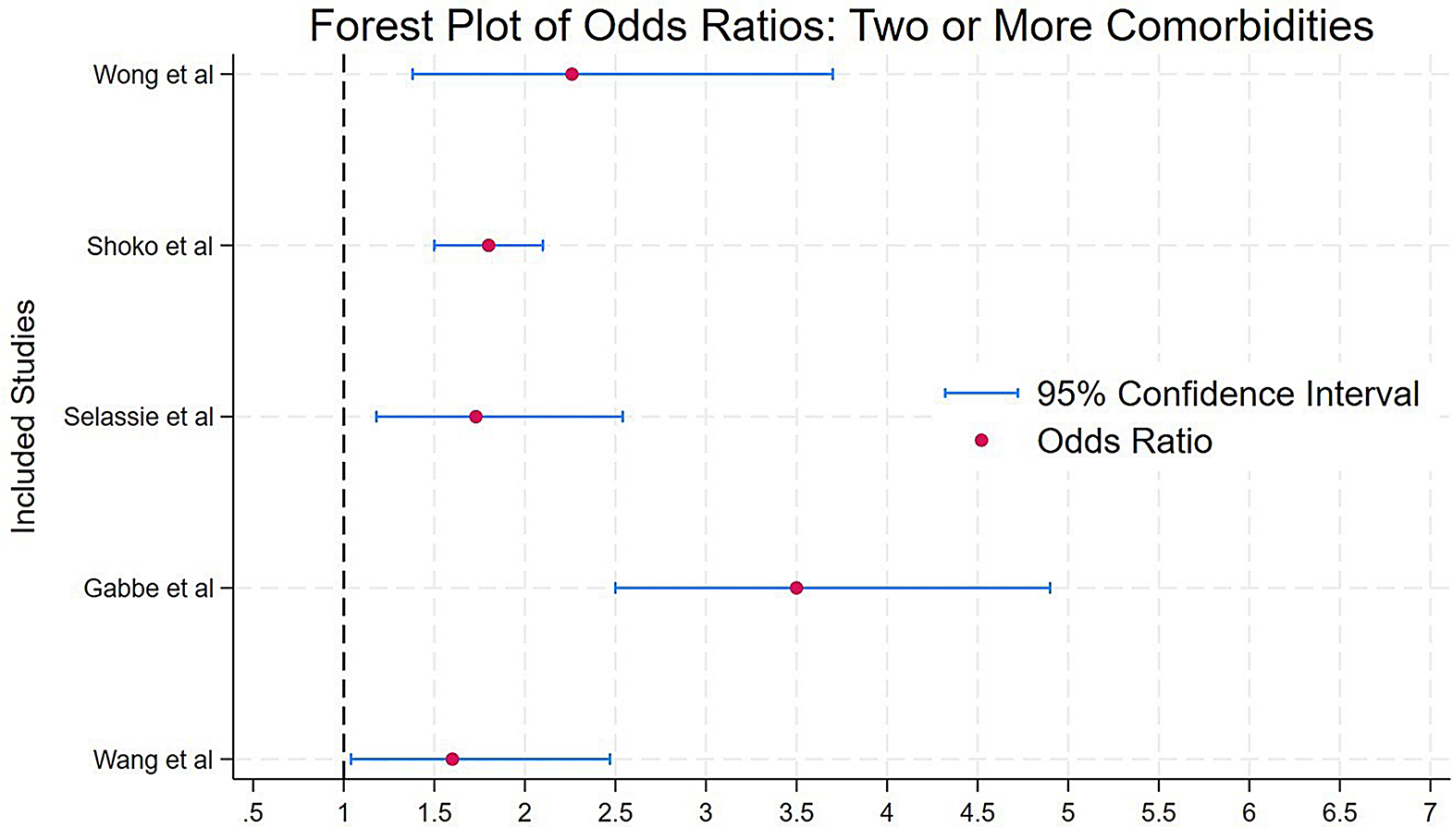
Forest plot of odds ratios (with 95% confidence intervals) for mortality in patients with two or more comorbidities, as reported in various studies **Shortlist of Explanations**: This forest plot displays odds ratios (OR) and 95% confidence intervals (CI) from studies examining mortality risk in trauma patients with two or more comorbidities. The vertical dashed line at OR = 1 represents the reference value (no effect). OR values to the right of this line (> 1) indicate an increased mortality risk, while values to the left (< 1) would suggest a decreased risk. All studies in this figure report OR values above 1, indicating a clear trend toward increased mortality risk in trauma patients with multiple comorbidities

A considerable number of studies examined the association between the number of comorbidities and subsequent case fatality with most reporting an increased risk as comorbidity burden increased. Figures 2 and 3 present the OR and confidence intervals (CI) for studies that reported case fatality with one comorbidity, and those with two or more comorbidities, respectively.

Studies assessing the impact of multiple comorbidities on trauma related case fatality found varying results. Selassie et al. reported that trauma patients with three or more pre-injury comorbidities had twice the comorbidity than those without comorbidities (OR 2.19; 95% CI 1.47–3.25; *p* < 0.0010) [34], Similarly Wong et al. found that patients with multiple comorbidities had over three times the odds of mortality (OR 3.36; 95% CI 1.98–5.69; *p* < 0.0010) [28]. However, Gabbe et al. found no significant association between comorbidities and case fatality (OR 1.1; 95% CI 0.2–4.4; *p* > 0.0500) [43].

While multiple studies reported an association between comorbidities and increased case fatality, some found no significant effect when evaluating individual comorbidities. Shoko et al. and Gabbe et al. found no difference in mortality rates when comparing trauma patients with one pre-injury comorbidity to those without [33, 43] Similarly, Selassie et al. specifically examined trauma patients with acute spinal cord injuries and found no clear impact of comorbidities on case fatality in this subgroup [34].

Blair et al. analysed 7,509 trauma patients and found that pre-injury comorbidities were associated with increased odds of mortality (OR 2.6; 95% CI 1.25–5.47; *p* < 0.0110). When stratified by ISS, patients with an ISS of 16–24 had higher case fatality if they had a pre-existing comorbidity compared to those without (OR 11.3 vs. 3.3; *p* < 0.0020) [26].

Niven et al. retrospectively analysed 3,080 trauma patients one year post-discharge and found that among those who died within a year, half had at least one pre-injury comorbidity (42 vs. 15%; *p* < 00010). Dementia and cerebrovascular disease accounted for 74% of these comorbidities, and each one-point increase in the CCI was associated with a 20% increased risk of delayed mortality (CI 1.02–1.51; *p* = 0.0300) [37].

Yiannoullou et al. found that trauma patients with a CCI score > 10, a score indicating significant comorbidity burden had an increased risk of case-fatality (OR 2.8; 95% CI 2.8–31.7; *p* < 0.0010) [49]. In contrast, de Vries et al. analysed polytrauma patients and found that pre-injury comorbidities did not significantly affect case fatality [50]. Passman et al.. found that trauma patients with pre-existing comorbidities had higher mortality rates at 30 day readmission compared to those without comorbidities [36]. Peng et al. found that among non-survivors with an unsurvivable ISS of 75 were more likely to have a pre-injury comorbidity (58 vs. 44%; *p* < 0.0001) [35].

Duvall et al. analysed 570,422 geriatric trauma patients, stratified into age groups 70–79 years and 80 years and older. They found that mortality increased with both age and injury severity, with patients aged 70–79 with an ISS between 40 and 49 having mortality rates between 42.6 and 45.5%, while those aged 80 and above had rates between 60.3 and 64.8% [51]. For an ISS of 50–59, mortality rates were 56.6–58.3% in the younger cohort and 71.9–76.1% in the older cohort. Among patients with an ISS of 66 mortality was 73.9% in the 70–79 age group and 93.3% in those aged 80 and above) [51]. Duvall et al. concluded that neither injury severity nor comorbidities alone could predict the futility of care in geriatric trauma patients. Additionally, they did not identify a specific ISS score with a mortality rate > 95% for any number of comorbidities in age group [51]. The highest mortality rate observed was in patients aged 80 and above with an ISS of 66 regardless of the presence of pre-existing comorbidities [51].

### Specific comorbidities

A substantial number of studies investigated the association specific pre-injury comorbidities and case fatality in major trauma patients. The most frequently studied comorbidities were cardiovascular diseases, diabetes, cancer, liver disease/cirrhosis, chronic kidney diseases, respiratory disease, coagulation disorders, hematological diseases and HIV. Table 2 presents the odds ratios for the impact of individual comorbidities on case fatality in major trauma patients.

**Table 2:** Reported odds ratios of studies of specific chronic conditions and their relation to mortality

Paper	Comorbidity	Odds Ratio	(95% confidence Intervals)	*p*-value
Wong et al.	Diabetes	1.31	1.68–2.52	0.04
Taylor et al.	Diabetes	1.25	0.99–1.59	0.065
Shu et al.	Diabetes	1.89	1.35–2.65	-
Brennan et al.	Diabetes	1.97*	0.96–4.03	-
Moore et al.	Diabetes	1.10	0.86–1.39	-
Shoko et al.	Diabetes	1.0	0.80–1.30	-
McGwin et al.	Diabetes	1.04	0.75–1.44	-
Wong et al.	Cancer	1.76	0.94–3.32	0.08
Wutzler et al.	Cancer	1.86	1.15-3.00	-
Shu et al.	Cancer	2.09	0.89–4.94	-
Toomey et al.	Cancer	2.58	1.91–3.49	< 0.001
Taylor et al.	Cancer	2.34	1.55–3.53	< 0.001
Shoko et al.	Cancer	3.40	1.90–6.20	-
Georgiou et al.	Chronic liver disease/cirrhosis	5.65	3.72–8.41	< 0.001
Wutzler et al.	Chronic liver disease/cirrhosis	2.18	1.32–3.58	-
Moore et al.	Chronic liver disease/cirrhosis	3.29	1.94–5.60	-
McGwin et al.	Chronic liver disease/cirrhosis	1.85*	1.13–3.04	-
Brennan et al.	Chronic liver disease/cirrhosis	11.11*	2.22–55.63	-
Shoko et al.	Chronic liver disease/cirrhosis	1.6	0.9–2.7	-
Pratt et al.	Chronic kidney disease	2.60	1.77–3.70	< 0.001
Moore et al.	Chronic kidney disease	2.66	1.86–3.79	-
McGwin et al.	Chronic kidney disease	2.42*	0.90–6.46	
Wutzler et al.	Cardiovascular disease	1.32	1.04–1.68	-
Taylor et al.	Cardiovascular disease	1.77	1.44–2.16	< 0.001
Brennan et al.	Cardiovascular disease	2.59*	1.24–5.41	-
Moore et al.	Cardiovascular disease	2.15	1.59–2.91	-
McGwin et al.	Cardiovascular disease	1.53*	1.32–1.77	-
Shoko et al.	Cardiovascular disease	1.3	0.80–1.90	-
Brennan et al.	Coagulation	1.59*	0.22–11.60	-
Moore et al.	Coagulation	2.85	1.94–4.19	-
Shoko et al.	Haematological	3.30	1.30–8.60	-
Brennan et al.	Respiratory disease	1.88*	0.90–3.92	-
McGwin et al.	Respiratory disease	0.75*	0.44–1.28	-
Taylor et al.	Respiratory disease	1.39	1.07–1.78	0.011
Shu et al.	Respiratory disease	1.52	0.76–3.04	-
Moore et al.	Respiratory disease	1.60	1.28–2.01	-
Shoko et al.	HIV infection	2.3	0.2–2.22	-

*Relative risk reported

Wong et al.. analysed 3,414 trauma patients and reported that pre-injury diabetes was associated with increased odds of case fatality at both one year and three-year (OR 1.3; 95% CI 1.7–2.5; *p* = 0.0400) [28]. Su et al. found that among 752 trauma patients, those with diabetic hyperglycemia had significantly higher mortality rates compared to those with stress induced hyperglycaemia(10.6 vs. 0.0%; *p* = 0.0220) or non-diabetic hyperglycaemia (5.3 vs. 0.0%; *p* = 0.0430) [45]. Shu et al. found that among trauma patients readmitted within 30 days post-hospitalisation, those with diabetes had approximately twice the odds of mortality (OR 1.9; 95% CI 1.4–2.7; *p* < 0.0010) while those with cardiovascular disease had almost six times the odds of mortality (OR 5.9; CI 4.7–7.4; *p* < 0.0010) [32]. Shoko et al. found that trauma patients with pre-existing peripheral vascular disease had significantly higher case fatality rates than those without (OR 2.6; 95% CI 1.8–3.6; *p* < 0.0010) [33].

### Question 4: does major trauma increase the risk of developing post-trauma comorbidities in the long-term?

Two studies examined the risk of developing comorbidities following major trauma. Stewart et al. examined 8,727 military service members from 2002 to 2016 and found that individuals who had sustained major trauma were at an increased risk of developing post-trauma hypertension (HR 2.78; 95% CI 2.18–3.55; *p* < 0.0010), diabetes mellitus (HR 4.45; CI 2.15–9.18; *p* < 0.0010) and coronary artery disease (HR 4.87; CI 2.11–11.25; *p* < 0.0010) compared to a non-injured cohort [52].

Gelaw et al. analysed data from 28,522 trauma patients between 2005 and 2018 comparing the prevalence of pre-injury, acute, and post-injury comorbidities. Before injury 15.4% of patients had at least one pre-existing comorbidity, increasing to 21% by five years post trauma. The most common new conditions were arthritis (20.7%), arthropathies (22.7%), cancer (14.7%) and cardiovascular disease (39.5%). No significant difference in comorbidity development was observed across different trauma types [10].

## Discussion

This scoping review examined the relationship between major trauma and comorbidities, focusing on both pre-injury comorbidities as predictors of trauma outcomes and the long term risk of developing comorbidities post trauma. Despite the extensive trauma literature, no previous systematic or scoping reviews have comprehensively explored these questions.

Several studies investigated the impact of pre-existing cardiovascular disease, diabetes, cancer, kidney disease and liver disease on trauma outcomes. The increasing life expectancy in high income countries, populations are living longer with multiple comorbidities, which presents challenges for managing these conditions in both trauma care and in the prevention of further complications [53]. This includes a higher risk of prolonged hospitalisation, increased complications, and greater resource utilisation in trauma management. This complexity necessitates coordinated inpatient management, often involving multiple medical and surgical teams further increasing the burden of disease [53, 54].

Traditionally, trauma has been associated with external causes such as motor vehicle accidents, falls and interpersonal violence [55]. However, demographic shifts suggest a growing burden of trauma among older adults, many of whom have pre-existing comorbidities [7]. This evolving epidemiology calls for adaptive trauma management approaches that integrate acute surgical interventions with enhanced medical care [56]. Co-management models involving both medical and surgical teams may play an increasingly critical role in optimising outcomes for these patients [56].

The absence of studies examining whether comorbidities increase susceptibility to major trauma may stem from several factors. Traditionally trauma research has focused on injury mechanisms, acute management, and outcomes, with less emphasis on pre-existing health conditions as risk factors. Establishing a direct causal link between specific comorbidities and trauma incidence is methodologically challenging, as trauma events result from a complex interplay of factors, including environmental hazards, polypharmacy and underlying disease related impairments. Existing studies on comorbidities in trauma populations primarily focus on post-injury outcomes such as mortality and morbidity, rather than on how these conditions might predispose individuals to traumatic events [29]. For example, while studies have demonstrated that a higher comorbidity burden is associated with increased hospital mortality, there remains a paucity of literature exploring whether specific pre-existing conditions directly contribute to trauma risk [29].

A novel aspect of this review was the consideration of comorbidities as potential risk factors for major trauma. It is plausible that certain medical conditions contribute to an increased likelihood of traumatic events. For example, vascular disease may predispose individuals to road trauma due to stroke-related impairments whilst driving. Similarly, polypharmacy – particularly sedatives, antihypertensives or hypoglycemic agents can lead to dizziness, falls or delayed reaction times, thereby increasing trauma risk. Neurological conditions such as Parkinson’s disease or cognitive impairment due to dementia may also increase susceptibility to injury by reducing situational awareness and coordination.

While falls in older adults and related risk management strategies are well documented, proactive trauma prevention strategies to individuals with comorbid conditions remain an area for further development [57]. Targeted education programs for patients and caregivers could enhance awareness of modifiable risk factors, such as medication management and mobility safety. Additionally, pre-hospital care protocols could be adapted to better identify and manage trauma patients with complex comorbidities upon first contact, ensuring that comorbidity related complications are anticipated early in care. Finally, rehabilitation programs should integrate multidisciplinary approaches that address both the acute recovery phase and long-term disease management to improve functional outcomes and reduce hospital readmissions. Given the growing burden of trauma in aging populations, the most significant gains in both quality of life and resource efficiency are likely to come from a combination of early interventions strategies and post-trauma care models that holistically address both injury and pre-existing health conditions.

Another key consideration is the possibility that traumatic injuries themselves contribute to the subsequent development of comorbidities. While it is well established that individuals with a traumatic brain injury are at increased risk of psychological comorbidities [58, 59], whether similar associations exist between other forms of major trauma and long term comorbidities remains unclear. It is plausible that injuries such as acute kidney injury could predispose individuals to chronic kidney disease, cardiac contusions could contribute to heart failure, and lung trauma could result in fibrosis or chronic respiratory conditions. Similarly, fractures and musculoskeletal injuries may increase the risk of osteoarthritis and chronic pain, while severe abdominal trauma could predispose individuals to intestinal adhesions or chronic bowel obstruction. Despite these plausible associations, the literature exploring long term comorbidity development after major trauma remains limited, with only two studies identified in our search. These studies reported an increased risk of developing hypertension, diabetes, cardiovascular disease, arthritis and cancer in trauma patients, particularly within the first five years post-injury [10, 52].

While this scoping review is the first to systematically explore the interplay between comorbidities and major trauma, several limitations warrant discussion. First individual comorbidities were not searched independently due to the large volume of literature that would have resulted. Second, our search was limited to academic databases and did not include international repositories, grey literature, or non-English studies without available translations, potentially limiting the breadth of included evidence. Third, consistent with the JBI scoping review methodology, we did not assess the quality or risk of bias of the included studies [8].

Additionally, this review excluded frailty and psychological conditions, such as insomnia and PTSD, despite their recognized role as risk factors for trauma. Frailty, as a multidimensional syndrome, is typically assessed using dedicated frailty indices rather than conventional comorbidity scoring system such as the CCI or APACHE making its inclusion outside the scope of this review [60]. Psychological conditions were excluded due to the difficulty in distinguishing pre-existing disorders from those that develop post-trauma, as well as their frequent classification under quality of life and functional outcomes rather than medical comorbidities [61]. Consequently, dementia which is included as part of the CCI, was not comprehensively examined [16]. Future research should explore the impact of frailty and psychological conditions on trauma outcomes, particularly in aging populations where these factors are increasingly relevant.

## Conclusion

This scoping review highlights gaps in the existing literature regarding the interplay between major trauma and comorbidities. As population age and comorbidity prevalence increases, trauma-related morbidity and mortality will continue to have a significant burden on healthcare systems. Further research is warranted to better characterise the role of pre-existing comorbidities in trauma risk and outcomes, as well as the potential for major trauma to contribute to the development of comorbidities. A more detailed understanding of these relationships could inform preventative strategies, optimise patient management and improve long term outcomes for trauma survivors.

## Electronic supplementary material

Below is the link to the electronic supplementary material.

Supplementary Material 1

Supplementary Material 2

Supplementary Material 3

## Data Availability

No datasets were generated or analysed during the current study.

## Declarations

### Protocol registration

https://osf.io/4ypr9.
