# Supplementary material for: Major trauma and comorbidity: a scoping review
Source: Eur J Trauma Emerg Surg. 2025 Mar 12;51(1):133. doi: 10.1007/s00068-025-02805-x (PMC11903538; doi:10.1007/s00068-025-02805-x)
Supplement: Supplementary file 2 — Supplementary Material 2 [file 68_2025_2805_MOESM2_ESM.docx]

# Appendix I: Search strategy

| **Embase (Elsevier)** |  |  |
| --- | --- | --- |
| **Search** | **Search terms** | **Number of hits** |
| #1 | chronic disease'/exp | 207,252 |
| #2 | (**'chronic'** NEXT/3 (**condition*** OR **diseas*** OR **diagnos*** OR **illness*** OR **disorder***)):ti,ab,kw | 513,357 |
| #3 | comorbidity'/exp | 296,749 |
| #4 | multiple chronic conditions'/exp | 4,634 |
| #5 | multimorbid*':ti,ab,kw OR 'co morbid':ti,ab,kw OR comorbid:ti,ab,kw OR 'multi-morbid':ti,ab,kw OR 'multiple comorbid*':ti,ab,kw OR 'multiple co-morbid*':ti,ab,kw | 97,907 |
| #6 | (('concurrent chronic' NEXT/3 (condition* OR diseas* OR diagnos* OR illness* OR disorder*)):ti,ab,kw) | 220 |
| #7 | (('simultaneous chronic' NEXT/3 (condition* OR diseas* OR diagnos* OR illness* OR disorder*)):ti,ab,kw) | 11 |
| #8 | ((number NEXT/3 ('co-morbid*' OR comorbid*)):ti,ab,kw) | 6,702 |
| #9 | (('multiple chronic' NEXT/3 (condition* OR diseas* OR diagnos* OR illness* OR disorder*)):ti,ab,kw) | 2,934 |
| #10 | charlson comorbidity index' | 26,116 |
| #11 | elixhauser:ti,ab,kw | 1,917 |
| #12 | pre existing conditions':ti,ab,kw | 1,015 |
| #13 | ('injur*' NEAR/1 (major OR trauma* OR critical OR 'life-threatening' OR 'life threatening' OR multiple)):ti,ab,kw | 26,247 |
| #14 | ('trauma*' NEAR/1 (major OR critical OR 'life-threatening' OR 'life threatening' OR multiple)):ti,ab,kw | 11,543 |
| #15 | #1 OR #2 OR #3 OR #4 OR #5 OR #6 OR #7 OR #8 OR #9 OR #10 OR #11 OR #12 | 975,361 |
| #16 | #13 OR #14 | 36,183 |
| #17 | #15 AND #16 | 1,392 |
| #18 | #17 AND [embase]/lim NOT ([embase]/lim AND [medline]/lim) | 512 |
|  |  |  |
| **Cochrane** |  |  |
| **Search** | **Search terms** | **Number of hits** |
| #1 | MeSH descriptor: [Chronic Disease] explode all trees | 13,356 |
| #2 | ('chronic' NEXT/3 (condition* OR diseas* OR diagnos* OR illness* OR disorder*)):ti,ab,kw | 58,264 |
| #3 | MeSH descriptor: [Comorbidity] explode all trees | 3,623 |
| #4 | MeSH descriptor: [Comorbidity] explode all trees | 33 |
| #5 | "multimorbid*":ti,ab,kw OR "co morbid":ti,ab,kw OR comorbid:ti,ab,kw OR "multi-morbid":ti,ab,kw OR "multiple comorbid*":ti,ab,kw OR "multiple co-morbid*":ti,ab,kw | 8,063 |
| #6 | (("concurrent chronic" NEXT/3 (condition* OR diseas* OR diagnos* OR illness* OR disorder*)):ti,ab,kw) | 1,595 |
| #7 | (("simultaneous chronic" NEXT/3 (condition* OR diseas* OR diagnos* OR illness* OR disorder*)):ti,ab,kw) | 1,595 |
| #8 | ((number NEXT/3 ("co-morbid*" OR comorbid*)):ti,ab,kw) | 23,998 |
| #9 | (("multiple chronic" NEXT/3 (condition* OR diseas* OR diagnos* OR illness* OR disorder*)):ti,ab,kw) | 352 |
| #10 | "charlson comorbidity index" | 1,595 |
| #11 | elixhauser:ti,ab,kw | 25 |
| #12 | ('trauma*' NEAR/1 (major OR critical OR "life-threatening" OR "life threatening" OR multiple)):ti,ab,kw | 835 |
| #13 | ('injur*' NEAR/1 (major OR trauma* OR critical OR 'life-threatening' OR 'life threatening' OR multiple)):ti,ab,kw | 2,942 |
| #14 | #1 OR #2 OR #3 OR #4 OR #5 OR #6 OR #7 OR #8 OR #9 OR #10 OR #11 | 81,172 |
| #15 | #12 OR #13 | 2,942 |
| #16 | #14 AND #15 | 170 |
| **CINAHL (EbscoHost)** |  |  |
| **Search** | **Search terms** | **Number of hits** |
| #1 | (MH "chronic disease") | 65,898 |
| #2 | AB ( ('chronic' W3 (condition* OR diseas* OR diagnos* OR illness* OR disorder*)) ) OR TI ( ('chronic' W3 (condition* OR diseas* OR diagnos* OR illness* OR disorder*)) ) | 108,056 |
| #3 | (MH "Comorbidity") | 64,326 |
| #4 | AB "multiple chronic conditions" OR TI "multiple chronic conditions" | 860 |
| #5 | AB ( "multimorbid*" OR "co morbid" OR "comorbid" OR "multi-morbid" OR "multiple comorbid*" OR "multiple co-morbid*" ) OR TI ( "multimorbid*" OR "co morbid" OR "comorbid" OR "multi-morbid" OR "multiple comorbid*" OR "multiple co-morbid*" ) | 24,256 |
| #6 | AB ( ( (("concurrent chronic" W3 (condition* OR diseas* OR diagnos* OR illness* OR disorder*)) ) ) OR TI ( ( (("concurrent chronic" W3 (condition* OR diseas* OR diagnos* OR illness* OR disorder*)) ) ) | 45 |
| #7 | AB ( (("simultaneous chronic" W3 (condition* OR diseas* OR diagnos* OR illness* OR disorder*)) ) ) OR TI ( (("simultaneous chronic" W3 (condition* OR diseas* OR diagnos* OR illness* OR disorder*)) ) ) | 3 |
| #8 | AB ( ((number W3 ("co-morbid*" OR comorbid*)) ) OR TI ( ((number W3 ("co-morbid*" OR comorbid*)) ) | 1,649 |
| #9 | AB ( (("multiple chronic" W3 (condition* OR diseas* OR diagnos* OR illness* OR disorder*)) ) OR TI ( (("multiple chronic" W3 (condition* OR diseas* OR diagnos* OR illness* OR disorder*)) ) | 1,314 |
| #10 | "charlson comorbidity index" | 2,461 |
| #11 | AB elixhauser OR TI elixhauser | 364 |
| #12 | AB "pre existing conditions" OR TI "pre existing conditions" | 228 |
| #13 | AB ( ( ("injur*" N1 (major OR trauma* OR critical OR "life-threatening" OR "life threatening" OR multiple)) ) OR TI ( ( ("injur*" N1 (major OR trauma* OR critical OR "life-threatening" OR "life threatening" OR multiple)) ) | 27,171 |
| #14 | AB ( ("trauma*" N1 (major OR critical OR "life-threatening" OR "life threatening" OR multiple)) ) OR TI ( ("trauma*" N1 (major OR critical OR "life-threatening" OR "life threatening" OR multiple)) ) | 3,763 |
| #15 | S1 OR S2 OR S3 OR S4 OR S5 OR S6 OR S7 OR S8 OR S9 OR S10 OR S11 OR S12 | 223,838 |
| #16 | S13 OR S14 | 30,025 |
| #17 | S15 AND S16 | 1,313 |
| **Medline (EbscoHost)** |  |  |
| **Search** | **Search terms** | **Number of hits** |
| #1 | (MH "chronic disease") | 267,058 |
| #2 | AB ( ( ('chronic' W3 (condition* OR diseas* OR diagnos* OR illness* OR disorder*)) ) OR TI ( ( ('chronic' W3 (condition* OR diseas* OR diagnos* OR illness* OR disorder*)) ) | 353,498 |
| #3 | (MH "Comorbidity") | 114,165 |
| #4 | (MH "Multiple Chronic Conditions") | 514 |
| #5 | AB ( ( "multimorbid*" OR "co morbid" OR "comorbid" OR "multi-morbid" OR "multiple comorbid*" OR "multiple co-morbid*" ) ) OR TI ( ( "multimorbid*" OR "co morbid" OR "comorbid" OR "multi-morbid" OR "multiple comorbid*" OR "multiple co-morbid*" ) ) | 61,023 |
| #6 | AB ( ( (("concurrent chronic" W3 (condition* OR diseas* OR diagnos* OR illness* OR disorder*)) ) ) OR TI ( ( (("concurrent chronic" W3 (condition* OR diseas* OR diagnos* OR illness* OR disorder*)) ) ) | 131 |
| #7 | AB ( (("simultaneous chronic" W3 (condition* OR diseas* OR diagnos* OR illness* OR disorder*)) ) ) OR TI ( (("simultaneous chronic" W3 (condition* OR diseas* OR diagnos* OR illness* OR disorder*)) ) ) | 4 |
| #8 | AB ( ((number W3 ("co-morbid*" OR comorbid*)) ) OR TI ( ((number W3 ("co-morbid*" OR comorbid*)) ) | 3,993 |
| #9 | AB ( (("multiple chronic" W3 (condition* OR diseas* OR diagnos* OR illness* OR disorder*)) ) OR TI ( (("multiple chronic" W3 (condition* OR diseas* OR diagnos* OR illness* OR disorder*)) ) | 2,061 |
| #10 | "charlson comorbidity index" | 6,630 |
| #11 | AB elixhauser OR TI elixhauser | 718 |
| #12 | AB "pre existing conditions" OR TI "pre existing conditions" | 659 |
| #13 | AB ( ( ("injur*" N1 (major OR trauma* OR critical OR "life-threatening" OR "life threatening" OR multiple)) ) OR TI ( ( ("injur*" N1 (major OR trauma* OR critical OR "life-threatening" OR "life threatening" OR multiple)) ) | 71,204 |
| #14 | AB ( ( ("trauma*" N1 (major OR critical OR "life-threatening" OR "life threatening" OR multiple)) ) OR TI ( ( ("trauma*" N1 (major OR critical OR "life-threatening" OR "life threatening" OR multiple)) ) | 10,413 |
| #15 | S1 OR S2 OR S3 OR S4 OR S5 OR S6 OR S7 OR S8 OR S9 OR S10 OR S11 OR S12 | 709,032 |
| #16 | S13 OR S14 | 79,211 |
| #17 | S15 AND S16 | 2,822 |
| **PubMed** |  |  |
| **Search** | **Search terms** | **Number of hits** |
| #1 | (((((chronic disease[MeSH Terms]) OR ("chronic disease"[Title/Abstract])) OR (Chronic condition[Title/Abstract])) OR (chronic diagnosis[Title/Abstract])) OR (chronic illness[Title/Abstract])) OR (chronic disorder[Title/Abstract]) | 308,447 |
| #2 | ((((("multiple chronic conditions"[Title/Abstract]) OR (multimorbid[Title/Abstract])) OR ("co morbid"[Title/Abstract])) OR (comorbid[Title/Abstract])) OR (multi-morbid[Title/Abstract])) OR (comorbidity[MeSH Terms]) | 153,996 |
| #3 | (((((((("Major injury"[Title/Abstract]) OR ("traumatic injury"[Title/Abstract])) OR ("traumatic injuries"[Title/Abstract])) OR ("critical injuries"[Title/Abstract])) OR ("life-threatening injuries"[Title/Abstract])) OR ("life-threatening injury"[Title/Abstract])) OR ("life threatening injury"[Title/Abstract])) OR ("life threatening injuries"[Title/Abstract])) OR ("multiple injuries"[Title/Abstract]) | 15,432 |
| #4 | (((((multiple trauma[MeSH Terms]) OR ("Major trauma"[Title/Abstract])) OR ("critical trauma"[Title/Abstract])) OR ("life-threatening trauma"[Title/Abstract])) OR ("life threatening trauma"[Title/Abstract])) OR ("Major trauma"[Title/Abstract]) | 16,692 |
| #5 | ((charlson comorbidity[Title/Abstract]) | 7,461 |
| #6 | #3 OR #4 | 30,465 |
| #7 | #1 OR #2 OR #5 | 458,868 |
| #8 | #6 OR #7 | 631 |
