# Supplementary material for: Major trauma and comorbidity: a scoping review
Source: Eur J Trauma Emerg Surg. 2025 Mar 12;51(1):133. doi: 10.1007/s00068-025-02805-x (PMC11903538; doi:10.1007/s00068-025-02805-x)
Supplement: Supplementary file 3 — Supplementary Material 3 [file 68_2025_2805_MOESM3_ESM.docx]

# Appendix II: Studies ineligible following full-text review

| **Reference** | **Reason for exclusion** |
| --- | --- |
| Pugh MJ, Finley EP, Wang C-P, Copeland LA, Jaramillo CA, Swan AA, et al. A retrospective cohort study of comorbidity trajectories associated with traumatic brain injury in veterans of the Iraq and Afghanistan wars. Brain Injury. 2016;30(12):1481-90. | Chronic pain |
| Holtslag HR, Post MW, Lindeman E, Van der Werken C. Long-term functional health status of severely injured patients. Injury. 2007 Mar 1;38(3):280-9. | Functional outcomes |
| Kumar RG, Ketchum JM, Corrigan JD, Hammond FM, Sevigny M, Dams-O’Connor K. The Longitudinal Effects of Co-morbid Health Burden on Functional Outcomes for Adults with Moderate-to-Severe Traumatic Brain Injury. The Journal of head trauma rehabilitation. 2020 Jul;35(4):E372. | Functional outcomes |
| Lewis FD, Horn GJ. Traumatic brain injury: analysis of functional deficits and posthospital rehabilitation outcomes. J Spec Oper Med. 2013 Jan 1;13(3):56-61. | Functional outcomes |
| Tan AL, Chiong Y, Nadkarni N, Cheng JY, Chiu MT, Wong TH. Predictors of Change in Functional Outcome at six months and twelve months after Severe Injury: A Retrospective Cohort Study. World Journal of Emergency Surgery. 2018 Dec;13:1-8. | Functional outcomes |
| Tee JW, Chan PC, Fitzgerald MC, Liew SM, Rosenfeld JV. Early predictors of functional disability after spine trauma: a level 1 trauma center study. Spine. 2013 May 20;38(12):999-1007. | Functional outcomes |
| Whiteneck GG, Ketchum JM, Almeida EJ, Goldstein R, Brown AW, Corrigan JD, Hammond FM, Weintraub AH, Tefertiller C. Developing an index of medical conditions associated with outcomes after moderate-to-severe traumatic brain injury. Journal of neurotrauma. 2021 Mar 1;38(5):593-603. | Functional outcomes |
| James A, Tran VT, Gauss T, Hamada S, Roquet F, Bitot V, Boutonnet M, Raux M, Ravaud P. Important issues to severe trauma survivors: a qualitative study. Annals of surgery. 2022 Jan 25;275(1):189-95. | Functional outcomes |
| Min L, Ubhayakar N, Saliba D, Kelley‐Quon L, Morley E, Hiatt J, Cryer H, Tillou A. The Vulnerable Elders Survey‐13 predicts hospital complications and mortality in older adults with traumatic injury: A pilot study. Journal of the American Geriatrics Society. 2011 Aug;59(8):1471-6. | Functional outcomes |
| Robbins GT, Goldstein R, Siddiqui S, Huang DS, Zafonte R, Schneider JC. Capture rates of comorbidity measures at inpatient rehabilitation facilities after a stroke or brain injury. PM&R. 2022 Apr;14(4):462-71. | Functional outcomes |
| van Erp S, Holtslag HR, van Beeck EF. Determinants of limitations in unpaid work after major trauma: a prospective cohort study with 15 months follow-up. Injury. 2014 Mar 1;45(3):629-34. | Functional outcomes |
| Corrigan JD, Zheng T, Pinto SM, Bogner J, Kean J, Niemeier JP, Guerrier TP, Haaland B, Horn SD. Effect of preexisting and co-occurring comorbid conditions on recovery in the 5 years after rehabilitation for traumatic brain injury. The Journal of Head Trauma Rehabilitation. 2020 May 1;35(3):E288-98. | Functional outcomes |
| Joyce JM, Monchi O, Ismail Z, Kibreab M, Cheetham J, Kathol I, Sarna J, Martino D, Debert CT. The impact of traumatic brain injury on cognitive and neuropsychiatric symptoms of Parkinson’s disease. International Review of Psychiatry. 2020 Jan 2;32(1):46-60 | Functional outcomes |
| Nguyen TQ, Simpson PM, Braaf SC, Gabbe BJ. Mortality, functional and return to work outcomes of major trauma patients injured from deliberate self-harm. Injury. 2017 Jan 1;48(1):184-94. | Functional outcomes |
| MacDonald SL, Robinson LR. An evaluation of age-based differences in the demographic features and clinical outcomes of trauma rehabilitation patients. American Journal of Physical Medicine & Rehabilitation. 2020 Nov 1;99(11):999-1003. | Functional outcomes |
| Sander AM, Roebuck TM, Struchen MA, Sherer M, High Jr WM. Long-term maintenance of gains obtained in postacute rehabilitation by persons with traumatic brain injury. The Journal of head trauma rehabilitation. 2001 Aug 1;16(4):356-73. | Functional outcomes |
| St Hilaire C, Johnson A, Loseth C, Alipour H, Faunce N, Kaminski S, Sharma R. Facial fractures after geriatric ground-level falls are a marker of functional decline and warrant trauma center admission. The American Surgeon. 2020 Oct;86(10):1302-6. | Isolated extremity injury |
| Chao LL. The relationship between traumatic brain injury and rates of chronic symptomatic illness in 202 Gulf War veterans. Military medicine. 2018 Nov 5;183(11-12):e571-9. | Psychiatric conditions |
| Saverino C, Swaine B, Jaglal S, Lewko J, Vernich L, Voth J, Calzavara A, Colantonio A. Rehospitalization after traumatic brain injury: a population-based study. Archives of physical medicine and rehabilitation. 2016 Feb 1;97(2):S19-25. | Psychiatric conditions |
| Yang KC, Zhou MJ, Sperry JL, Rong L, Zhu XG, Geng L, Wu W, Zhao G, Billiar TR, Feng QM. Significant sex-based outcome differences in severely injured Chinese trauma patients. Shock. 2014 Jul 1;42(1):11-5. | Comorbidities not separately analysed. |
| Tee JW, Chan PC, Gruen RL, Fitzgerald MC, Liew SM, Cameron PA, Rosenfeld JV. Early predictors of mortality after spine trauma: a level 1 Australian trauma center study. Spine. 2013 Jan 15;38(2):169-77. | Comorbidities not separately analysed. |
| Tran A, Mai T, El-Haddad J, Lampron J, Yelle JD, Pagliarello G, Matar M. Preinjury ASA score as an independent predictor of readmission after major traumatic injury. Trauma surgery & acute care open. 2017 Nov 1;2(1):e000128. | Comorbidities not separately analysed. |
| Sheehan M, Brent L, Deasy C. Trauma on Farms in the Republic of Ireland. Injury. 2020 Sep 1;51(9):2025-32. | Comorbidities not separately analysed |
| Aas RW, Haveraaen LA, Brouwers EP, Skarpaas LS. Who among patients with acquired brain injury returned to work after occupational rehabilitation? The rapid-return-to-work-cohort-study. Disability and rehabilitation. 2018 Oct 9;40(21):2561-70. | Mild to moderate TBI only |
| Lee RY, Brumback LC, Sathitratanacheewin S, Lober WB, Modes ME, Lynch YT, Ambrose CI, Sibley J, Vranas KC, Sullivan DR, Engelberg RA. Association of physician orders for life-sustaining treatment with ICU admission among patients hospitalized near the end of life. Jama. 2020 Mar 10;323(10):950-60. | Mild to moderate TBI only |
| Lee YK, Lee CW, Huang MY, Hsu CY, Su YC. Increased risk of ischemic stroke in patients with mild traumatic brain injury: a nationwide cohort study. Scandinavian journal of trauma, resuscitation and emergency medicine. 2014 Dec;22:1-7. | Mild to moderate TBI only |
| Lucas S, Smith BM, Temkin N, Bell KR, Dikmen S, Hoffman JM. Comorbidity of headache and depression after mild traumatic brain injury. Headache: The Journal of Head and Face Pain. 2016 Feb;56(2):323-30. | Mild to moderate TBI only |
| Schmidt BR, Moos RM, Könü-Leblebicioglu D, Bischoff-Ferrari HA, Simmen HP, Pape HC, Neuhaus V. Higher age is a major driver of in-hospital adverse events independent of comorbid diseases among patients with isolated mild traumatic brain injury. European Journal of Trauma and Emergency Surgery. 2019 Apr 1;45:191-8. | Mild to moderate TBI only |
| Sercy E, Orlando A, Carrick M, Lieser M, Madayag R, Vasquez D, Tanner A, Rubin B, Bar-Or D. Long-term mortality and causes of death among patients with mild traumatic brain injury: a 5-year multicenter study. Brain Injury. 2020 Mar 20;34(4):556-66. | Mild to moderate TBI only |
| Vanderploeg RD, Curtiss G, Luis CA, Salazar AM. Long-term morbidities following self-reported mild traumatic brain injury. Journal of clinical and experimental neuropsychology. 2007 Aug 6;29(6):585-98. | Mild to moderate TBI only |
| James SL, Theadom A, Ellenbogen RG, Bannick MS, Montjoy-Venning W, Lucchesi LR, Abbasi N, Abdulkader R, Abraha HN, Adsuar JC, Afarideh M. Global, regional, and national burden of traumatic brain injury and spinal cord injury, 1990–2016: a systematic analysis for the Global Burden of Disease Study 2016. The Lancet Neurology. 2019 Jan 1;18(1):56-87. | No chronic illness |
| Aldrian S, Nau T, Koenig F, Vécsei V. Geriatric polytrauma. Wiener klinische Wochenschrift. 2005 Feb;117:145-9. | No chronic illness |
| Bakhshayesh P, Weidenhielm L, Enocson A. Factors affecting mortality and reoperations in high-energy pelvic fractures. European journal of orthopaedic surgery & traumatology. 2018 Oct;28:1273-82. | No chronic illness |
| Barr LV, Vindlacheruvu M, Gooding CR. The effect of becoming a major trauma centre on outcomes for elderly hip fracture patients. Injury. 2015 Feb 1;46(2):384-7. | No chronic illness |
| Barra ME, Izzy S, Sarro-Schwartz A, Hirschberg RE, Mazwi N, Edlow BL. Stimulant therapy in acute traumatic brain injury: prescribing patterns and adverse event rates at 2 level 1 trauma centers. Journal of intensive care medicine. 2020 Nov;35(11):1196-202. | No chronic illness |
| Baum J, Entezami P, Shah K, Medhkour A. Predictors of outcomes in traumatic brain injury. World neurosurgery. 2016 Jun 1;90:525-9. | No chronic illness |
| Bayer J, Lefering R, Reinhardt S, Kühle J, Zwingmann J, Südkamp NP, Hammer T, TraumaRegister DGU. Thoracic trauma severity contributes to differences in intensive care therapy and mortality of severely injured patients: analysis based on the TraumaRegister DGU®. World journal of emergency surgery. 2017 Dec;12:1-9. | No chronic illness |
| Brandt CP, Yowler CJ, Fratianne RB. Burns with multiple trauma. The american surgeon. 2002 Mar;68(3):240-4. | No chronic illness |
| Brooks SE, Mukherjee K, Gunter OL, Guillamondegui OD, Jenkins JM, Miller RS, May AK. Do models incorporating comorbidities outperform those incorporating vital signs and injury pattern for predicting mortality in geriatric trauma?. Journal of the American College of Surgeons. 2014 Nov 1;219(5):1020-7. | No chronic illness |
| Brown AW, Leibson CL, Mandrekar J, Ransom JE, Malec JF. Long-term survival after traumatic brain injury: a population-based analysis controlled for nonhead trauma. The Journal of head trauma rehabilitation. 2014 Jan;29(1):E1. | No chronic illness |
| Caufeild J, Singhal A, Moulton R, Brenneman F, Redelmeier D, Baker AJ. Trauma recidivism in a large urban Canadian population. Journal of Trauma and Acute Care Surgery. 2004 Oct 1;57(4):872-6. | No chronic illness |
| DiFazio LT, Curran T, Bilaniuk JW, Adams JM, Durling-Grover R, Kong K, Nemeth ZH. The impact of the COVID-19 pandemic on hospital admissions for trauma and acute care surgery. The American Surgeon. 2020 Aug;86(8):901-3. | No chronic illness |
| Dijkink S, van der Wilden GM, Krijnen P, Dol L, Rhemrev S, King DR, DeMoya MA, Velmahos GC, Schipper IB. Polytrauma patients in the Netherlands and the USA: a bi-institutional comparison of processes and outcomes of care. Injury. 2018 Jan 1;49(1):104-9. | No chronic illness |
| Gowing R, Jain MK. Injury patterns and outcomes associated with elderly trauma victims in Kingston, Ontario. Canadian journal of surgery. 2007 Dec;50(6):437. | No chronic illness |
| Green RS, Butler MB, Erdogan M. Increased mortality in trauma patients who develop postintubation hypotension. Journal of Trauma and Acute Care Surgery. 2017 Oct 1;83(4):569-74. | No chronic illness |
| Harrison-Felix C, Kolakowsky-Hayner SA, Hammond FM, Wang R, Englander J, Dams-O'Connor K, Kreider SE, Novack TA, Diaz-Arrastia R. Mortality after surviving traumatic brain injury: risks based on age groups. The Journal of head trauma rehabilitation. 2012 Nov 1;27(6):E45-56. | No chronic illness |
| Jacobsson LJ, Westerberg M, Lexell J. Demographics, injury characteristics and outcome of traumatic brain injuries in northern Sweden. Acta Neurologica Scandinavica. 2007 Nov;116(5):300-6. | No chronic illness |
| Kwon M, Lekoubou A, Bishu KG, Ovbiagele B. Association of seizure co-morbidity with early hospital readmission among traumatic brain injury patients. Brain injury. 2020 Oct 14;34(12):1625-9. | No chronic illness |
| Llompart-Pou JA, Chico-Fernández M, Sánchez-Casado M, Alberdi-Odriozola F, Guerrero-López F, Mayor-García MD, González-Robledo J, Ballesteros-Sanz MÁ, Herrán-Monge R, León-López R, López-Amor L. Age-related injury patterns in Spanish trauma ICU patients. Results from the RETRAUCI. Injury. 2016 Sep 1;47:S61-5. | No chronic illness |
| Lorelli DR, Kralovich KA, Seguin C. The impact of pre-existing end-stage renal disease on survival in acutely injured trauma patients. The American surgeon. 2001 Jul;67(7):693-6. | No chronic illness |
| Omar M, Moore L, Lauzier F, Tardif PA, Dufresne P, Boutin A, Lessard-Bonaventure P, Paquet J, Clément J, Turgeon AF. Complications following hospital admission for traumatic brain injury: a multicenter cohort study. Journal of critical care. 2017 Oct 1;41:1-8. | No chronic illness |
| Pearson WS, Sugerman DE, McGuire LC, Coronado VG. Emergency department visits for traumatic brain injury in older adults in the United States: 2006-08. Western Journal of Emergency Medicine: Integrating Emergency Care with Population Health. 2012;13(3). | No chronic illness |
| Reinhold M, Knop C, Beisse R, Audige L, Kandziora F, Pizanis A, Pranzl R, Gercek E, Schultheiss M, Weckbach A, Buehren V. Operative treatment of 733 patients with acute thoracolumbar spinal injuries: comprehensive results from the second, prospective, Internet-based multicenter study of the Spine Study Group of the German Association of Trauma Surgery. European Spine Journal. 2010 Oct;19:1657-76. | No chronic illness |
| Salvo F, Luppi F, Lucchesi DM, Canovi S, Franchini S, Polese A, Santi F, Trabucco L, Fasano T, Ferrari AM. Serum Copeptin levels in the emergency department predict major clinical outcomes in adult trauma patients. BMC emergency medicine. 2020 Dec;20(1):1-0. | No chronic illness |
| Scotti P, Séguin C, Lo BW, De Guise E, Troquet JM, Marcoux J. Antithrombotic agents and traumatic brain injury in the elderly population: hemorrhage patterns and outcomes. Journal of neurosurgery. 2019 Jul 5;133(2):486-95. | No chronic illness |
| Shekhar C, Gupta LN, Premsagar IC, Sinha M, Kishore J. An epidemiological study of traumatic brain injury cases in a trauma centre of New Delhi (India). Journal of emergencies, trauma, and shock. 2015 Jul;8(3):131. | No chronic illness |
| Trentzsch H, Lefering R, Nienaber U, Kraft R, Faist E, Piltz S. The role of biological sex in severely traumatized patients on outcomes: a matched-pair analysis. Annals of surgery. 2015 Apr 1;261(4):774-80. | No chronic illness |
| Vallier HA, Super DM, Moore TA, Wilber JH. Do patients with multiple system injury benefit from early fixation of unstable axial fractures? vThe effects of timing of surgery on initial hospital course. Journal of orthopaedic trauma. 2013 Jul 1;27(7):405-12. | No chronic illness |
| Verma V, Singh A, Singh GK, Kumar S, Sharma V, Kumar A, Kumar V. Epidemiology of trauma victims admitted to a level 2 trauma center of North India. International journal of critical illness and injury science. 2017 Apr;7(2):107. | No chronic illness |
| Winkler EA, Yue JK, Burke JF, Chan AK, Dhall SS, Berger MS, Manley GT, Tarapore PE. Adult sports-related traumatic brain injury in United States trauma centers. Neurosurgical focus. 2016 Apr 1;40(4):E4. | No chronic illness |
| Wong E, Fong YT. Trauma airway experience by emergency physicians. European Journal of Emergency Medicine. 2003 Sep 1;10(3):209-12. | No chronic illness |
| Yadollahi M, Arabi AH, Mahmoudi A, Zamani M, Farahmand M. Blunt thoracic injury mortality and clinical presentation. Trauma Monthly. 2018 Jul 1;23(4). | No chronic illness |
| Caplan B, Bogner J, Brenner L, Malec J, Kumar RG, Juengst SB, Wang Z, Dams-O'Connor K, Dikmen SS, O'Neil-Pirozzi TM, Dahdah MN. Epidemiology of comorbid conditions among adults 50 years and older with traumatic brain injury. Journal of head trauma rehabilitation. 2018 Jan 1;33(1):15-24. | No major trauma TBI only |
| Caplan B, Bogner J, Brenner L, Malec J, Kumar RG, Juengst SB, Wang Z, Dams-O'Connor K, Dikmen SS, O'Neil-Pirozzi TM, Dahdah MN. Epidemiology of comorbid conditions among adults 50 years and older with traumatic brain injury. Journal of head trauma rehabilitation. 2018 Jan 1;33(1):15-24. | No major trauma TBI only |
| Chen YH, Keller JJ, Kang JH, Lin HC. Association between traumatic brain injury and the subsequent risk of brain cancer. Journal of neurotrauma. 2012 May 1;29(7):1328-33. | No major trauma TBI only |
| Chou YC, Yeh CC, Hu CJ, Meng NH, Chiu WT, Chou WH, Chen TL, Liao CC. Risk and mortality of traumatic brain injury in stroke patients: two nationwide cohort studies. The Journal of Head Trauma Rehabilitation. 2014 Nov 1;29(6):514-21. | No major trauma TBI only |
| Colantonio A, Ratcliff G, Chase S, Vernich L. Aging with traumatic brain injury: long-term health conditions. International Journal of Rehabilitation Research. 2004 Sep 1;27(3):209-14. | No major trauma TBI only |
| Coronado VG, Thomas KE, Sattin RW, Johnson RL. The CDC traumatic brain injury surveillance system: characteristics of persons aged 65 years and older hospitalized with a TBI. The Journal of head trauma rehabilitation. 2005 May 1;20(3):215-28. | No major trauma TBI only |
| Cusimano MD, Saarela O, Hart K, Zhang S, McFaull SR. A population-based study of fall-related traumatic brain injury identified in older adults in hospital emergency departments. Neurosurgical focus. 2020 Oct 1;49(4):E20. | No major trauma TBI only |
| Donohue JT, Clark DE, DeLorenzo MA. Long-term survival of Medicare patients with head injury. Journal of Trauma and Acute Care Surgery. 2007 Feb 1;62(2):419-23. | No major trauma TBI only |
| Ferguson PL, Smith GM, Wannamaker BB, Thurman DJ, Pickelsimer EE, Selassie AW. A population‐based study of risk of epilepsy after hospitalization for traumatic brain injury. Epilepsia. 2010 May;51(5):891-8. | No major trauma TBI only |
| Fu TS, Jing R, McFaull SR, Cusimano MD. Recent trends in hospitalization and in-hospital mortality associated with traumatic brain injury in Canada: a nationwide, population-based study. Journal of Trauma and Acute Care Surgery. 2015 Sep 1;79(3):449-55. | No major trauma TBI only |
| Gebregziabher M, Ward RC, Taber DJ, Walker RJ, Ozieh M, Dismuke CE, Axon RN, Egede LE. Ethnic and geographic variations in multimorbidty: evidence from three large cohorts. Social Science & Medicine. 2018 Aug 1;211:198-206. | No major trauma TBI only |
| Hammond FM, Corrigan JD, Ketchum JM, Malec JF, Dams-O’Conner K, Hart T, Novack TA, Bogner J, Dahdah MN, Whiteneck GG. Prevalence of medical and psychiatric comorbidities following traumatic brain injury. The Journal of head trauma rehabilitation. 2019 Jul;34(4):E1. | No major trauma TBI only |
| Heiden SM, Caldwell BS. Considerations for developing chronic care system for traumatic brain injury based on comparisons of cancer survivorship and diabetes management care. Ergonomics. 2018 Jan 2;61(1):134-47. | No major trauma TBI only |
| Holcomb EM, Millis SR, Hanks RA. Comorbid disease in persons with traumatic brain injury: descriptive findings using the modified cumulative illness rating scale. Archives of physical medicine and rehabilitation. 2012 Aug 1;93(8):1338-42. | No major trauma TBI only |
| Holmes M, Garver M, Albrecht L, Arbabi S, Pham TN. Comparison of two comorbidity scoring systems for older adults with traumatic injuries. Journal of the American College of Surgeons. 2014 Oct 1;219(4):631-7. | No major trauma TBI only |
| Kumar RG, Olsen J, Juengst SB, Dams-O'Connor K, O'Neil-Pirozzi TM, Hammond FM, Wagner AK. Comorbid conditions among adults 50 years and older with traumatic brain injury: examining associations with demographics, healthcare utilization, institutionalization, and 1-year outcomes. The Journal of head trauma rehabilitation. 2019 Jul 1;34(4):224-32. | No major trauma TBI only |
| Liao CC, Chou YC, Yeh CC, Hu CJ, Chiu WT, Chen TL. Stroke risk and outcomes in patients with traumatic brain injury: 2 nationwide studies. InMayo Clinic Proceedings 2014 Feb 1 (Vol. 89, No. 2, pp. 163-172). Elsevier. | No major trauma TBI only |
| Liao JC, Ho CH, Liang FW, Wang JJ, Lin KC, Chio CC, Kuo JR. One-year mortality associations in hemodialysis patients after traumatic brain injury—an eight-year population-based study. PLoS One. 2014 Apr 8;9(4):e93956. | No major trauma TBI only |
| Lustenberger T, Talving P, Lam L, Inaba K, Bass M, Plurad D, Demetriades D. Effect of diabetes mellitus on outcome in patients with traumatic brain injury: a national trauma databank analysis. Brain Injury. 2013 Mar 1;27(3):281-5. | No major trauma TBI only |
| Manchester K, Corrigan JD, Singichetti B, Huang L, Bogner J, Yi H, Yang J. Current health status and history of traumatic brain injury among Ohio adults. Injury prevention. 2020 Apr 1;26(2):129-37. | No major trauma TBI only |
| Marwitz JH, Cifu DX, Englander J, High Jr WM. A multi-center analysis of rehospitalizations five years after brain injury. The Journal of head trauma rehabilitation. 2001 Aug 1;16(4):307-17. | No major trauma TBI only |
| Nyam TT, Ho CH, Wang YL, Lim SW, Wang JJ, Chio CC, Kuo JR, Wang CC. The risk of traumatic brain injury occurring among patients with Parkinson disease: a 14-year population-based study. World neurosurgery. 2018 May 1;113:e328-35. | No major trauma TBI only |
| Rau CS, Kuo PJ, Wu SC, Chen YC, Hsieh HY, Hsieh CH. Association between the osteoporosis self-assessment tool for asians score and mortality in patients with isolated moderate and severe traumatic brain injury: a propensity score-matched analysis. International Journal of Environmental Research and Public Health. 2016 Dec;13(12):1203 | No major trauma TBI only |
| Rau CS, Wu SC, Chen YC, Chien PC, Hsieh HY, Kuo PJ, Hsieh CH. Stress-induced hyperglycemia, but not diabetic hyperglycemia, is associated with higher mortality in patients with isolated moderate and severe traumatic brain injury: analysis of a propensity score-matched population. International journal of environmental research and public health. 2017 Nov;14(11):1340. | No major trauma TBI only |
| Seifi A, Dengler B, Martinez P, Godoy DA. Pulmonary embolism in severe traumatic brain injury. Journal of Clinical Neuroscience. 2018 Nov 1;57:46-50. | No major trauma TBI only |
| Selassie AW, Cao Y, Church EC, Saunders LL, Krause J. Accelerated death rate in population-based cohort of persons with traumatic brain injury. The Journal of head trauma rehabilitation. 2014 May 1;29(3):E8-19. | No major trauma TBI only |
| Selassie AW, McCarthy ML, Ferguson PL, Tian J, Langlois JA. Risk of posthospitalization mortality among persons with traumatic brain injury, South Carolina 1999–2001. The Journal of head trauma rehabilitation. 2005 May 1;20(3):257-69. | No major trauma TBI only |
| Svedung Wettervik T, Lenell S, Enblad P, Lewén A. Pre-injury antithrombotic agents predict intracranial hemorrhagic progression, but not worse clinical outcome in severe traumatic brain injury. Acta neurochirurgica. 2021 May;163:1403-13. | No major trauma TBI only |
| Wasfie T, Maxwell J, Parsons A, Hille J, Yapchai R, Hella J, Cwalina N, Barber KR, Shapiro B. Traumatic brain injury in the elderly: can we reduce readmissions? A community hospital experience. The American Surgeon. 2020 Dec;86(12):1647-50. | No major trauma TBI only |
| Wee HY, Ho CH, Chang CH, Chio CC, Wang JJ, Wang CC, Kuo JR. Probability of new-onset cancer between patients with traumatic brain injury and a comparison general population cohort. World Neurosurgery. 2019 Jan 1;121:e817-26. | No major trauma TBI only |
| Wilson DA, Selassie AW. Risk of severe and repetitive traumatic brain injury in persons with epilepsy: A population-based case–control study. Epilepsy & Behavior. 2014 Mar 1;32:42-8. | No major trauma TBI only |
| Yue JK, Robinson CK, Winkler EA, Upadhyayula PS, Burke JF, Pirracchio R, Suen CG, Deng H, Ngwenya LB, Dhall SS, Manley GT. Circadian variability of the initial Glasgow Coma Scale score in traumatic brain injury patients. Neurobiology of sleep and circadian rhythms. 2017 Jan 1;2:85-93. | No major trauma TBI only |
| Yuguero O, Guzman M, Castañ T, Forné C, Galindo G, Pujol J. Characteristics and prognosis of patients admitted to a hospital emergency department for traumatic brain injury and with anticoagulant or antiplatelet treatment. Neurocirugía (English Edition). 2018 Sep 1;29(5):233-9. | No major trauma TBI only |
| Whiteman C, Davidov DM, Sikora R, Paulson D, Schaefer G. Major trauma and the elder West Virginian: a six year review at a level I trauma center. The West Virginia medical journal. 2016 May;112(3):94. | No ISS>15 or ICU Admin |
| Gowing R, Jain MK. Injury patterns and outcomes associated with elderly trauma victims in Kingston, Ontario. Canadian journal of surgery. 2007 Dec;50(6):437. | No ISS>15 or ICU Admin |
| Zietlow SP, Capizzi PJ, Bannon MP, Farnell MB. Multisystem geriatric trauma. Journal of Trauma and Acute Care Surgery. 1994 Dec 1;37(6):985-8. | No ISS>15 or ICU Admin |
| Aamer A, Bin Zahid A, Shabbir J, Patel R, Chagla D, Das M. Impact of Comprehensive Geriatric Assessment on community dwelling elders: in an inner city hospital population. InJOURNAL OF THE AMERICAN GERIATRICS SOCIETY 2020 Apr 1 (Vol. 68, pp. S198-S199). 111 RIVER ST, HOBOKEN 07030-5774, NJ USA: WILEY. | No ISS>15 or ICU Admin |
| Baker PN, Salar O, Ollivere BJ, Forward DP, Weerasuriya N, Moppett IK, Moran CG. Evolution of the hip fracture population: time to consider the future? A retrospective observational analysis. BMJ open. 2014 Apr 1;4(4):e004405. | No ISS>15 or ICU Admin |
| Bible JE, Kadakia RJ, Wegner A, Richards JE, Mir HR. One-year mortality after isolated pelvic fractures with posterior ring involvement in elderly patients. Orthopedics. 2013 Jun 1;36(6):760-4. | No ISS>15 or ICU Admin |
| Brawer AE, Goel N. The onset of rheumatoid arthritis following trauma. Open Access Rheumatology: Research and Reviews. 2016 Aug 17:77-80. | No ISS>15 or ICU Admin |
| Chan V, Mollayeva T, Ottenbacher KJ, Colantonio A. Clinical profile and comorbidity of traumatic brain injury among younger and older men and women: a brief research notes. BMC research notes. 2017 Dec;10(1):1-7. | No ISS>15 or ICU Admin |
| Charles EJ, Napoli NJ, Johnston LE, Foster CA, Goode DA, Parker TB, Sharp EA, Barnes L, Young JS. Outcomes after falls continue to worsen despite trauma and geriatric care advancements. The American Surgeon. 2018 Mar;84(3):392-7. | No ISS>15 or ICU Admin |
| Cox S, Roggenkamp R, Bernard S, Smith K. The epidemiology of elderly falls attended by emergency medical services in Victoria, Australia. Injury. 2018 Sep 1;49(9):1712-9. | No ISS>15 or ICU Admin |
| Dai YT, Wu SC, Weng R. Unplanned hospital readmission and its predictors in patients with chronic conditions. Journal of the Formosan Medical Association. 2002 Nov 1;101(11):779-85. | No ISS>15 or ICU Admin |
| Destefano C, Shaw K, Stewart I, Poltavskiy E, Chung K, Walker L, Boggs N, Suo Y, Gundlapalli A. Does Trauma Heighten the Risk of Hematologic Malignancies? a Retrospective Study of US Combat Veterans. Blood. 2019 Nov 13;134:4130. | No ISS>15 or ICU Admin |
| Earl-Royal EC, Kaufman EJ, Hanlon AL, Holena DN, Rising KL, Delgado MK. Factors associated with hospital admission after an emergency department treat and release visit for older adults with injuries. The American journal of emergency medicine. 2017 Sep 1;35(9):1252-7. | No ISS>15 or ICU Admin |
| Fann JR, Ribe AR, Pedersen HS, Fenger-Grøn M, Christensen J, Benros ME, Vestergaard M. Long-term risk of dementia among people with traumatic brain injury in Denmark: a population-based observational cohort study. The Lancet Psychiatry. 2018 May 1;5(5):424-31. | No ISS>15 or ICU Admin |
| Fazel S, Wolf A, Pillas D, Lichtenstein P, Långström N. Suicide, fatal injuries, and other causes of premature mortality in patients with traumatic brain injury: a 41-year Swedish population study. JAMA psychiatry. 2014 Mar 1;71(3):326-33. | No ISS>15 or ICU Admin |
| Halvachizadeh S, Gröbli L, Berk T, Jensen KO, Hierholzer C, Bischoff-Ferrari HA, Pfeifer R, Pape HC. The effect of geriatric comanagement (GC) in geriatric trauma patients treated in a level 1 trauma setting: A comparison of data before and after the implementation of a certified geriatric trauma center. PLoS One. 2021 Jan 11;16(1):e0244554. | No ISS>15 or ICU Admin |
| Herrmann M, Curio N, Petz T, Synowitz H, Wagner S, Bartels C, Wallesch CW. Coping with illness after brain diseasesa comparison between patients with malignant brain tumors, stroke, Parkinson's disease and traumatic brain injury. Disability and rehabilitation. 2000 Jan 1;22(12):539-46. | No ISS>15 or ICU Admin |
| Himanen L, Portin R, Hämäläinen P, Hurme S, Hiekkanen H, Tenovuo O. Risk factors for reduced survival after traumatic brain injury: a 30-year follow-up study. Brain injury. 2011 May 1;25(5):443-52. | No ISS>15 or ICU Admin |
| Huang TH, Chen CZ, Kuo HI, Er HP, Lin SH. Enhanced risk of traumatic brain injury in patients with chronic obstructive pulmonary disease. Journal of investigative medicine. 2020 Apr;68(4):846-55. | No ISS>15 or ICU Admin |
| Jackson HM, Troeung L, Martini A. Prevalence, patterns, and predictors of multimorbidity in adults with acquired brain injury at admission to staged community-based rehabilitation. Archives of Rehabilitation Research and Clinical Translation. 2020 Dec 1;2(4):100089. | No ISS>15 or ICU Admin |
| Jacob L, Azouvi P, Kostev K. Age-related changes in the association between traumatic brain injury and dementia in older men and women. Journal of head trauma rehabilitation. 2021 May 13;36(3):E139-46. | No ISS>15 or ICU Admin |
| Jaffe MP, O'Neill J, Vandergoot D, Gordon WA, Small B. The unveiling of traumatic brain injury in an HIV/AIDS population. Brain injury. 2000 Jan 1;14(1):35-44. | No ISS>15 or ICU Admin |
| Jørgensen TS, Hansen AH, Sahlberg M, Gislason GH, Torp‐Pedersen C, Andersson C, Holm E. Nationwide time trends and risk factors for in‐hospital falls‐related major injuries. International journal of clinical practice. 2015 Jun;69(6):703-9. | No ISS>15 or ICU Admin |
| Jourdan C, Azouvi P, Genêt F, Selly N, Josseran L, Schnitzler A. Disability and health consequences of traumatic brain injury: national prevalence. American journal of physical medicine & rehabilitation. 2018 May 1;97(5):323-31. | No ISS>15 or ICU Admin |
| Kahn JM, Le T, Angus DC, Cox CE, Hough CL, White DB, Yende S, Carson SS. The epidemiology of chronic critical illness in the United States. Critical care medicine. 2015 Feb;43(2):282. | No ISS>15 or ICU Admin |
| Kornblith E, Peltz CB, Xia F, Plassman B, Novakovic-Apopain T, Yaffe K. Sex, race, and risk of dementia diagnosis after traumatic brain injury among older veterans. Neurology. 2020 Sep 29;95(13):e1768-75. | No ISS>15 or ICU Admin |
| LoBue C, Wilmoth K, Cullum CM, Rossetti HC, Lacritz LH, Hynan LS, Hart J, Womack KB. Traumatic brain injury history is associated with earlier age of onset of frontotemporal dementia. Journal of Neurology, Neurosurgery & Psychiatry. 2016 Aug 1;87(8):817-20. | No ISS>15 or ICU Admin |
| Loewenstern J, Kessler RA, Caridi J. Diabetes comorbidity increases risk of postoperative complications in traumatic thoracic vertebral fracture repair: a propensity score matched analysis. World Neurosurgery. 2019 Jan 1;121:e792-7. | No ISS>15 or ICU Admin |
| Long AF, Kneafsey R, Ryan J, Berry J. The role of the nurse within the multi‐professional rehabilitation team. Journal of advanced nursing. 2002 Jan;37(1):70-8. | No ISS>15 or ICU Admin |
| Lu K, Liang CL, Li PC, Liliang PC, Huang CY, Lee YC, Wang KW, Yang SN, Sun YT, Wang HK. Risk factors for myocardial dysfunction after traumatic brain injury: a one-year follow-up study. Injury. 2017 Aug 1;48(8):1794-800. | No ISS>15 or ICU Admin |
| McCann MR, Hill WF, Yan J, Rehou S, Jeschke MG. Burn injury and multiple sclerosis: A retrospective case-control study. Burns. 2019 Feb 1;45(1):247-52. | No ISS>15 or ICU Admin |
| Moore L, Stelfox HT, Turgeon AF, Nathens AB, Le Sage N, Émond M, Bourgeois G, Lapointe J, Gagné M. Rates, patterns, and determinants of unplanned readmission after traumatic injury: a multicenter cohort study. Annals of surgery. 2014 Feb 1;259(2):374-80. | No ISS>15 or ICU Admin |
| Nestvold K, Stavem K. Determinants of health-related quality of life 22 years after hospitalization for traumatic brain injury. Brain Injury. 2009 Jan 1;23(1):15-21. | No ISS>15 or ICU Admin |
| Onizuka N, Gaichas A, Roesler J. Hospital-Treated Falls and Comorbidities Among Older Adults in Minnesota, 2010-2014. Minnesota medicine. 2017 Mar 1;100(2):40-4. | No ISS>15 or ICU Admin |
| Pugh MJ, Finley EP, Copeland LA, Wang CP, Noel PH, Amuan ME, Parsons HM, Wells M, Elizondo B, Pugh JA. Complex comorbidity clusters in OEF/OIF veterans: the polytrauma clinical triad and beyond. Medical care. 2014 Feb 1:172-81. | No ISS>15 or ICU Admin |
| Regasa LE, Kaplan DA, Martin EM, Langbein J, Johnson F, Chase LC. Mortality Following Hospital Admission for US Active Duty Service Members Diagnosed With Penetrating Traumatic Brain Injury, 2004–2014. The Journal of Head Trauma Rehabilitation. 2018 Mar 1;33(2):123-32. | No ISS>15 or ICU Admin |
| Rehman NU, Dar MI, Bansal M, Kasliwal RR. Clinical outcomes of submassive pulmonary embolism thrombolysis—an Indian experience. The Egyptian Heart Journal. 2020 Dec;72:1-8. | No ISS>15 or ICU Admin |
| Salar O, Baker PN, Forward DP, Ollivere BJ, Weerasuriya N, Moppett IK, Moran CG. Predictors of direct home discharge following fractured neck of femur. The Annals of The Royal College of Surgeons of England. 2017 Jul;99(6):444-51. | No ISS>15 or ICU Admin |
| Saunders LL, Selassie AW, Hill EG, Horner MD, Nicholas JS, Lackland DT, Corrigan JD. Pre-existing health conditions and repeat traumatic brain injury. Archives of physical medicine and rehabilitation. 2009 Nov 1;90(11):1853-9. | No ISS>15 or ICU Admin |
| Scheid R, von Cramon DY. Clinical findings in the chronic phase of traumatic brain injury: data from 12 years’ experience in the Cognitive Neurology Outpatient Clinic at the University of Leipzig. Deutsches Arzteblatt International. 2010 Mar;107(12):199. | No ISS>15 or ICU Admin |
| Shi HY, Hwang SL, Lee KT, Lin CL. Temporal trends and volume-outcome associations after traumatic brain injury: a 12-year study in Taiwan. Journal of Neurosurgery. 2013 Apr 1;118(4):732-8. | No ISS>15 or ICU Admin |
| Silva MA, Calvo D, Brennan EM, Reljic T, Drasher-Phillips L, Schwartz DJ, Kumar A, Cotner BA, Taylor DJ, Nakase-Richardson R. Incidence and predictors of adherence to sleep apnea treatment in rehabilitation inpatients with acquired brain injury. Sleep Medicine. 2020 May 1;69:159-67. | No ISS>15 or ICU Admin |
| Sörbo A, Eiving I, Theodorsson E, Rydenhag B, Jonsdottir IH. Pre‐traumatic conditions can influence cortisol levels before and after a brain injury. Acta Neurologica Scandinavica. 2020 Apr;141(4):342-50. | No ISS>15 or ICU Admin |
| Swan AA, Amuan ME, Morissette SB, Finley EP, Eapen BC, Jaramillo CA, Pugh MJ. Long-term physical and mental health outcomes associated with traumatic brain injury severity in post-9/11 veterans: A retrospective cohort study. Brain injury. 2018 Dec 6;32(13-14):1637-50. | No ISS>15 or ICU Admin |
| Vande Walle N, Kenis C, Heeren P, Van Puyvelde K, Decoster L, Beyer I, Conings G, Flamaing J, Lobelle JP, Wildiers H, Milisen K. Fall predictors in older cancer patients: a multicenter prospective study. BMC geriatrics. 2014 Dec;14(1):1-0. | No ISS>15 or ICU Admin |
| Wang HK, Lin SH, Sung PS, Wu MH, Hung KW, Wang LC, Huang CY, Lu K, Chen HJ, Tsai KJ. Population based study on patients with traumatic brain injury suggests increased risk of dementia. Journal of Neurology, Neurosurgery & Psychiatry. 2012 Nov 1;83(11):1080-5. | No ISS>15 or ICU Admin |
| Winstead ML, Clegg DJ, Heidel RE, Ledderhof NJ, Gotcher JE. Fall-related facial trauma: A retrospective review of fracture patterns and medical comorbidity. Journal of Oral and Maxillofacial Surgery. 2021 Apr 1;79(4):864-70. | No ISS>15 or ICU Admin |
| No ISS>15 or ICU Admin Wu CL, Kor CT, Chiu PF, Tsai CC, Lian IB, Yang TH, Tarng DC, Chang CC. Long-term renal outcomes in patients with traumatic brain injury: A nationwide population-based cohort study. PLoS One. 2017 Feb 14;12(2):e0171999. | No ISS>15 or ICU Admin |
| Wu L, Cheng B. Analysis of perioperative risk factors for deep vein thrombosis in patients with femoral and pelvic fractures. Journal of Orthopaedic Surgery and Research. 2020 Dec;15(1):1-3. | No ISS>15 or ICU Admin |
| Xiang H, Kidwell K, Wheeler K. Role of disability in the association between obesity and unintentional injuries. Disability and health journal. 2008 Apr 1;1(2):99-107. | No ISS>15 or ICU Admin |
| Zivadinov R, Raj B, Ramanathan M, Teter B, Durfee J, Dwyer MG, Bergsland N, Kolb C, Hojnacki D, Benedict RH, Weinstock-Guttman B. Autoimmune comorbidities are associated with brain injury in multiple sclerosis. American Journal of Neuroradiology. 2016 Jun 1;37(6):1010-6. | No ISS>15 or ICU Admin |
| Adiamah A, Thompson A, Lewis-Lloyd C, Dickson E, Blackburn L, Moody N, Gida S, La Valle A, Reilly JJ, Saunders J, Brooks A. The ICON Trauma Study: the impact of the COVID-19 lockdown on major trauma workload in the UK. European journal of trauma and emergency surgery. 2021 Jun;47:637-45. | ISS not separately analysed |
| Álvarez EC, Castro AG, Lasa MO, Alfonso AJ, Acha PE, Borregán JR, Martín YP, Sotos TD. Epidemiology of traumatic brain injury in the elderly over a 25 year period. Revista Española de Anestesiología y Reanimación (English Edition). 2018 Dec 1;65(10):546-51. | ISS not separately analysed |
| Anandasivam NS, Russo GS, Fischer JM, Samuel AM, Ondeck NT, Swallow MS, Chung SH, Bohl DD, Grauer JN. Analysis of bony and internal organ injuries associated with 26,357 adult femoral shaft fractures and their impact on mortality. Orthopedics. 2017 May 1;40(3):e506-12. | ISS not separately analysed |
| Avcı A, Saraç EÖ, Eren TŞ, Onat S, Ülkü R, Özçelik C. Risk factors affecting post-traumatic acute respiratory distress syndrome development in thoracic trauma patients. Turkish Journal of Thoracic and Cardiovascular Surgery. 2019 Oct;27(4):540. | ISS not separately analysed |
| Battistella FD, Din AM, Perez L. Trauma patients 75 years and older: long-term follow-up results justify aggressive management. Journal of Trauma and Acute Care Surgery. 1998 Apr 1;44(4):618-24 | ISS not separately analysed |
| Bhattacharya B, Maung A, Schuster K, Davis KA. The older they are the harder they fall: injury patterns and outcomes by age after ground level falls. Injury. 2016 Sep 1;47(9):1955-9. | ISS not separately analysed |
| Brotemarkle RA, Resnick B, Michaels K, Morton P, Wells C. Length of hospital stay and discharge disposition in older trauma patients. Geriatric nursing. 2015 Mar 1;36(2):S3-9. | ISS not separately analysed |
| Calvo RY, Sise CB, Sise MJ, Bansal V. Quantifying the burden of pre-existing conditions in older trauma patients: A novel metric based on mortality risk. The American Journal of Emergency Medicine. 2019 Oct 1;37(10):1836-45. | ISS not separately analysed |
| Cameron CM, Purdie DM, Kliewer EV, McClure RJ. Ten-year outcomes following traumatic brain injury: a population-based cohort. Brain Injury. 2008 Jan 1;22(6):437-49. | ISS not separately analysed |
| Chalya PL, Mchembe M, Mabula JB, Kanumba ES, Gilyoma JM. Etiological spectrum, injury characteristics and treatment outcome of maxillofacial injuries in a Tanzanian teaching hospital. Journal of trauma management & outcomes. 2011 Dec;5(1):1-6. | ISS not separately analysed |
| Copertino LM, McCormack JE, Rutigliano DN, Huang EC, Shapiro MJ, Vosswinkel JA, Jawa RS. Early unplanned hospital readmission after acute traumatic injury: the experience at a state-designated level-I trauma center. The American Journal of Surgery. 2015 Feb 1;209(2):268-73. | ISS not separately analysed |
| Delahanty DL, Marley R, Fenton A, Salvator A, Woofter C, Erck D, Coleman J, Muakkassa F. A comparison between survival from cancer before and after a physical traumatic injury: physical trauma before cancer is associated with decreased survival. Journal of Trauma Management & Outcomes. 2015 Dec;9(1):1-9. | ISS not separately analysed |
| DiMaggio C, Ayoung-Chee P, Shinseki M, Wilson C, Marshall G, Lee DC, Wall S, Maulana S, Pachter HL, Frangos S. Traumatic injury in the United States: in-patient epidemiology 2000–2011. Injury. 2016 Jul 1;47(7):1393-403. | ISS not separately analysed |
| Evans DC, Gerlach AT, Christy JM, Jarvis AM, Lindsey DE, Whitmill ML, Eiferman D, Murphy CV, Cook CH, Beery PR, Steinberg SM. Pre-injury polypharmacy as a predictor of outcomes in trauma patients. International journal of critical illness and injury science. 2011 Jul;1(2):104. | ISS not separately analysed |
| Gardner J, Sexton KW, Taylor J, Beck W, Kimbrough MK, Davis B, Bhavaraju A, Karim S, Porter A. Defining severe traumatic brain injury readmission rates and reasons in a rural state. Trauma Surgery & Acute Care Open. 2018 Sep 1;3(1):e000186 | ISS not separately analysed |
| Grigorian A, Albertson S, Delaplain PT, Gabriel V, Maithel S, Dosch A, Schubl S, Joe V, Nahmias J. Cirrhosis increases complication rate and overall mortality in patients with traumatic lung injury. Trauma. 2019 Jul;21(3):201-7. | ISS not separately analysed |
| Hannan EL, Waller CH, Farrell LS, Rosati C. Elderly trauma inpatients in New York state: 1994–1998. Journal of Trauma and Acute Care Surgery. 2004 Jun 1;56(6):1297-304. | ISS not separately analysed |
| Ho KM, Burrell M, Rao S, Baker R. Incidence and risk factors for fatal pulmonary embolism after major trauma: a nested cohort study. British journal of anaesthesia. 2010 Nov 1;105(5):596-602. | ISS not separately analysed |
| Hui X, Haider AH, Hashmi ZG, Rushing AP, Dhiman N, Scott VK, Selvarajah S, Haut ER, Efron DT, Schneider EB. Increased risk of pneumonia among ventilated patients with traumatic brain injury: every day counts!. journal of surgical research. 2013 Sep 1;184(1):438-43. | ISS not separately analysed |
| Hwabejire JO, Kaafarani HM, Lee J, Yeh DD, Fagenholz P, King DR, de Moya MA, Velmahos GC. Patterns of injury, outcomes, and predictors of in-hospital and 1-year mortality in nonagenarian and centenarian trauma patients. JAMA surgery. 2014 Oct 1;149(10):1054-9. | ISS not separately analysed |
| Kahl JE, Calvo RY, Sise MJ, Sise CB, Thorndike JF, Shackford SR. The changing nature of death on the trauma service. Journal of Trauma and Acute Care Surgery. 2013 Aug 1;75(2):195-201. | ISS not separately analysed |
| Kao LS, Todd SR, Moore FA. The impact of diabetes on outcome in traumatically injured patients: an analysis of the National Trauma Data Bank. The American journal of surgery. 2006 Dec 1;192(6):710-4. | ISS not separately analysed |
| Kung SC, Lin WT, Tsai TC, Lin MH, Chang CH, Lai CC, Chao CM. Epidemiologic characteristics and outcomes of major trauma patients requiring prolonged mechanical ventilation. Medicine. 2017 Dec;96(52). | ISS not separately analysed |
| Küçük MP, Küçük AO, Aksoy İ, Aydın D, Ülger F. Prognostic evaluation of cases with thoracic trauma admitted to the intensive care unit: 10-year clinical outcomes. Turkish Journal of Trauma & Emergency Surgery/Ulusal Travma ve Acil Cerrahi Dergisi. 2019 Jan 1;25(1). | ISS not separately analysed |
| Mitchell RJ, Curtis K, Braithwaite J. Health outcomes and costs for injured young people hospitalised with and without chronic health conditions. Injury. 2017 Aug 1;48(8):1776-83. | ISS not separately analysed |
| Mitchell R, Harvey L, Stanford R, Close J. Health outcomes and costs of acute traumatic spinal injury in New South Wales, Australia. The Spine Journal. 2018 Jul 1;18(7):1172-9. | ISS not separately analysed |
| Monaghan SF, Heffernan DS, Thakkar RK, Reinert SE, Machan JT, Connolly MD, Gregg SC, Kozloff MS, Adams Jr CA, Cioffi WG. The development of a urinary tract infection is associated with increased mortality in trauma patients. Journal of Trauma and Acute Care Surgery. 2011 Dec 1;71(6):1569-74. | ISS not separately analysed |
| Nosanov LB, McLawhorn MM, Vigiola Cruz M, Chen JH, Shupp JW. A national perspective on ECMO utilization use in patients with burn injury. Journal of Burn Care & Research. 2017 Dec 27;39(1):10-4. | ISS not separately analysed |
| Opalek JM, Graymire VL, Redd D. Wheelchair falls: 5 years of data from a level I trauma center. Journal of Trauma Nursing\| JTN. 2009 Apr 1;16(2):98-102. | ISS not separately analysed |
| Osborn TM, Tracy JK, Dunne JR, Pasquale M, Napolitano LM. Epidemiology of sepsis in patients with traumatic injury. Critical care medicine. 2004 Nov 1;32(11):2234-40. | ISS not separately analysed |
| Ozmen O, Aksoy M, Ince I, Dostbil A, Dogan N, Kursad H. Comparing the clinical features and Trauma Scores of trauma patients aged under 65 years with those of patients aged over 65 years in the intensive care unit: a retrospective study for last ten years. The Eurasian Journal of Medicine. 2020 Feb;52(1):1. | ISS not separately analysed |
| Petrey LB, Weddle RJ, Richardson B, Gilder R, Reynolds M, Bennett M, Cook A, Foreman M, Warren AM. Trauma patient readmissions: Why do they come back for more?. Journal of Trauma and Acute Care Surgery. 2015 Nov 1;79(5):717-25. | ISS not separately analysed |
| Pollifrone M, Callender L, Bennett M, Driver S, Petrey L, Hamilton R, Dubiel R. Predictors for 30-day readmissions after traumatic brain injury. Journal of Head Trauma Rehabilitation. 2021 May 13;36(3):E178-85. | ISS not separately analysed |
| Rau CS, Wu SC, Chen YC, Chien PC, Hsieh HY, Kuo PJ, Hsieh CH. Effect of age on Glasgow Coma Scale in patients with moderate and severe traumatic brain injury: an approach with propensity score-matched population. International journal of environmental research and public health. 2017 Nov;14(11):1378. | ISS not separately analysed |
| Richmond TS, Kauder D, Strumpf N, Meredith T. Characteristics and outcomes of serious traumatic injury in older adults. Journal of the American Geriatrics Society. 2002 Feb;50(2):215-22. | ISS not separately analysed |
| Rosso A, Brazinova A, Janciak I, Wilbacher I, Rusnak M, Mauritz W. Severe traumatic brain injury in Austria II: epidemiology of hospital admissions. Wiener Klinische Wochenschrift. 2007 Feb 1;119(1-2):29-34. | ISS not separately analysed |
| Sacco WJ, Copes WS, Bain Jr LW, MacKenzie EJ, Frey CF, Hoyt DB, Weigelt JA, Champion HR. Effect of preinjury illness on trauma patient survival outcome. The Journal of trauma. 1993 Oct 1;35(4):538-42. | ISS not separately analysed |
| Saverino C, Swaine B, Jaglal S, Lewko J, Vernich L, Voth J, Calzavara A, Colantonio A. Rehospitalization after traumatic brain injury: a population-based study. Archives of physical medicine and rehabilitation. 2016 Feb 1;97(2):S19-25. | ISS not separately analysed |
| Scheetz LJ. Injury patterns, severity and outcomes among older adults who sustained brain injury following a same level fall: a retrospective analysis. International emergency nursing. 2015 Apr 1;23(2):162-7. | ISS not separately analysed |
| Shibahashi K, Sugiyama K, Okura Y, Hoda H, Hamabe Y. Multicenter retrospective cohort study of “talk and die” after traumatic brain injury. World Neurosurgery. 2017 Nov 1;107:82-6. | ISS not separately analysed |
| Stawicki SP, Hoff WS, Hoey BA, Grossman MD, Scoll B, Reed III JF. Human immunodeficiency virus infection in trauma patients: where do we stand?. Journal of Trauma and Acute Care Surgery. 2005 Jan 1;58(1):88-93. | ISS not separately analysed |
| Sumiyoshi K, Hayakawa T, Yatsushige H, Shigeta K, Momose T, Enomoto M, Sato S, Takasato Y. Outcome of traumatic brain injury in patients on antiplatelet agents: a retrospective 20-year observational study in a single neurosurgery unit. Brain injury. 2017 Sep 19;31(11):1445-54. | ISS not separately analysed |
| Tan CP, Ng A, Civil I. Co-morbidities in trauma patients: common and significant. The New Zealand Medical Journal (Online). 2004 Sep 10;117(1201). | ISS not separately analysed |
| Tebby J, Lecky F, Edwards A, Jenks T, Bouamra O, Dimitriou R, Giannoudis PV. Outcomes of polytrauma patients with diabetes mellitus. BMC medicine. 2014 Dec;12:1-1. | ISS not separately analysed |
| Thompson HJ, Rivara FP, Nathens A, Wang J, Jurkovich GJ, Mackenzie EJ. Development and validation of the mortality risk for trauma comorbidity index. Annals of surgery. 2010 Aug;252(2):370. | ISS not separately analysed |
| Thompson HJ, Dikmen S, Temkin N. Prevalence of comorbidity and its association with traumatic brain injury and outcomes in older adults. Research in gerontological nursing. 2012 Jan 1;5(1):17-24. | ISS not separately analysed |
| Vanzant EL, Hilton RE, Lopez CM, Zhang J, Ungaro RF, Gentile LF, Szpila BE, Maier RV, Cuschieri J, Bihorac A, Leeuwenburgh C. Advanced age is associated with worsened outcomes and a unique genomic response in severely injured patients with hemorrhagic shock. Critical care. 2015 Dec;19(1):1-5. | ISS not separately analysed |
| Ventura T, Harrison-Felix C, Carlson N, DiGuiseppi C, Gabella B, Brown A, DeVivo M, Whiteneck G. Mortality after discharge from acute care hospitalization with traumatic brain injury: a population-based study. Archives of physical medicine and rehabilitation. 2010 Jan 1;91(1):20-9. | ISS not separately analysed |
| Yang M, Hayward RD, Edhayan E. Associations between cardiovascular comorbidities and mortality, length of hospital stay, and total charges among traumatic injury patients. European journal of trauma and emergency surgery. 2021 Jun;47:861-7. | ISS not separately analysed |
| Yen CI, Chiou MJ, Kuo CF, Liao HT. Determination of risk factors for burn mortality based on a regional population study in Taiwan. Burns. 2018 Sep 1;44(6):1591-601. | ISS not separately analysed |
| Adam N, Sorensen V, Skinner R. Not all intestinal traumatic injuries are the same: A comparison of surgically treated blunt vs. penetrating injuries. Injury. 2015 Jan 1;46(1):115-8. | Irrelevant |
| Allan BJ, Davis JS, Pandya RK, Jouria J, Habib F, Namias N, Schulman CI. Exploring trauma recidivism in an elderly cohort. Journal of surgical research. 2013 Sep 1;184(1):582-5. | Irrelevant |
| Ao KH, Ho CH, Wang CC, Wang JJ, Chio CC, Kuo JR. The increased risk of stroke in early insomnia following traumatic brain injury: a population-based cohort study. Sleep medicine. 2017 Sep 1;37:187-92. | Irrelevant |
| Ávila Martínez RJ, Hernández Voth A, Marrón Fernández C, Hermoso Alarza F, Martínez Serna I, Mariscal de Alba A, Zuluaga Bedoya M, Trujillo MD, Meneses Pardo JC, Díaz Hellin V, Larru Cabrero E, Gámez García AP. Evolution and complications of chest trauma. Arch Bronconeumol. 2013 May;49(5):177-80. | Irrelevant |
| Bobeff EJ, Fortuniak J, Bryszewski B, Wiśniewski K, Bryl M, Kwiecień K, Stawiski K, Jaskólski DJ. Mortality after traumatic brain injury in elderly patients: a new scoring system. World neurosurgery. 2019 Aug 1;128:e129-47. | Irrelevant |
| Bohl DD, Ondeck NT, Samuel AM, Diaz-Collado PJ, Nelson SJ, Basques BA, Leslie MP, Grauer JN. Demographics, mechanisms of injury, and concurrent injuries associated with calcaneus fractures: a study of 14 516 patients in the American College of Surgeons National Trauma Data Bank. Foot & Ankle Specialist. 2017 Oct;10(5):402-10. | Irrelevant |
| Boutin A, Moore L, Lauzier F, Chassé M, English S, Zarychanski R, McIntyre L, Griesdale D, Fergusson DA, Turgeon AF. Transfusion of red blood cells in patients with traumatic brain injuries admitted to Canadian trauma health centres: a multicentre cohort study. BMJ open. 2017 Mar 1;7(3):e014472. | Irrelevant |
| Brinkhof MW, Al-Khodairy A, Eriks-Hoogland I, Fekete C, Hinrichs T, Hund-Georgiadis M, Meier S, Scheel-Sailer A, Schubert M, Reinhardt JD. Health conditions in people with spinal cord injury: contemporary evidence from a population-based community survey in Switzerland. Journal of rehabilitation medicine. 2016 Feb;48(2):197-209. | Irrelevant |
| Brown SB, Colantonio A, Kim H. Gender differences in discharge destination among older adults following traumatic brain injury. Health care for women international. 2012 Oct 1;33(10):896-904. | Irrelevant |
| Bukur M, Teurel C, Catino J, Kurek S. The price of always saying yes: a cost analysis of secondary overtriage to an urban level I trauma center. The American Surgeon. 2018 Aug;84(8):1368-75. | Irrelevant |
| Camacho‐Soto A, Warden MN, Searles Nielsen S, Salter A, Brody DL, Prather H, Racette BA. Traumatic brain injury in the prodromal period of Parkinson's disease: A large epidemiological study using medicare data. Annals of neurology. 2017 Nov;82(5):744-54. | Irrelevant |
| Caplan B, Bogner J, Brenner L, Kumar RG, Boles JA, Wagner AK. Chronic inflammation after severe traumatic brain injury: characterization and associations with outcome at 6 and 12 months postinjury. Journal of Head Trauma Rehabilitation. 2015 Nov 1;30(6):369-81. | Irrelevant |
| Caplan B, Bogner J, Brenner L, Simpson GK, Daher M, Hodgkinson A, Strettles B. Comparing the injury profile, service use, outcomes, and comorbidities of people with severe TBI across urban, regional, and remote populations in New South Wales: a multicentre study. Journal of head trauma rehabilitation. 2016 Mar 1;31(2):E26-38. | Irrelevant |
| Castriotta RJ, Wilde MC, Lai JM, Atanasov S, Masel BE, Kuna ST. Prevalence and consequences of sleep disorders in traumatic brain injury. Journal of Clinical Sleep Medicine. 2007 Jun 15;3(4):349-56. | Irrelevant |
| Chaudhry R, Batra S, Mancillas OL, Wegner R, Grewal N, Williams GW. In-hospital mortality with use of percutaneous endoscopic gastrostomy in traumatic brain injury patients: results of a nationwide population-based study. Neurocritical care. 2017 Apr;26:232-8. | Irrelevant |
| Chen SF, Su YC, Wang LY, Hsu CY, Shen YC. Tourette's syndrome is associated with an increased risk of traumatic brain injury: A nationwide population-based cohort study. Parkinsonism & Related Disorders. 2019 Jun 1;63:88-93. | Irrelevant |
| Christensen MC, Parr M, Tortella BJ, Malmgren J, Morris S, Rice T, Holcomb JB, CONTROL Study Group. Global differences in causes, management, and survival after severe trauma: the recombinant activated factor VII phase 3 trauma trial. Journal of Trauma and Acute Care Surgery. 2010 Aug 1;69(2):344-52. | Irrelevant |
| Colantonio A, Gerber G, Bayley M, Deber R, Yin J, Kim H. Differential profiles for patients with traumatic and non-traumatic brain injury. | Irrelevant |
| Cox S, Morrison C, Cameron P, Smith K. Advancing age and trauma: triage destination compliance and mortality in Victoria, Australia. Injury. 2014 Sep 1;45(9):1312-9. | Irrelevant |
| Dias MD, Fontes B, Poggetti RS, Birolini D. Hyperbaric oxygen therapy: types of injury and number of sessions-a review of 1506 cases. Undersea & Hyperbaric Medicine. 2008;35(1):53. | Irrelevant |
| Dams-O'Connor K, Spielman L, Singh A, Gordon WA, Lingsma HF, Maas AI, Manley GT, Mukherjee P, Okonkwo DO, Puccio AM, Schnyer DM. The impact of previous traumatic brain injury on health and functioning: a TRACK-TBI study. Journal of neurotrauma. 2013 Dec 15;30(24):2014-20. | Irrelevant |
| Denson K, Morgan D, Cunningham R, Nigliazzo A, Brackett D, Lane M, Smith B, Albrecht R. Incidence of venous thromboembolism in patients with traumatic brain injury. The American journal of surgery. 2007 Mar 1;193(3):380-4. | Irrelevant |
| Dunham CM, Watson LA, Cooper C. Base deficit level indicating major injury is increased with ethanol. The Journal of emergency medicine. 2000 Feb 1;18(2):165-71. | Irrelevant |
| Efron DT, Sorock G, Haut ER, Chang D, Schneider E, MacKenzie E, Cornwell III EE, Jurkovich GJ. Preinjury statin use is associated with improved in-hospital survival in elderly trauma patients. Journal of Trauma and Acute Care Surgery. 2008 Jan 1;64(1):66-74. | Irrelevant |
| Enumah S, Lilley EJ, Nitzschke S, Haider AH, Salim A, Cooper Z. Increased hospice enrollment and decreased neurosurgical interventions without changes in mortality for older Medicare patients with moderate to severe traumatic brain injury. The American Journal of Surgery. 2018 Jun 1;215(6):1016-9. | Irrelevant |
| Ferrah N, Cameron P, Gabbe B, Fitzgerald M, Martin K, Beck B. Trends in the nature and management of serious abdominal trauma. World journal of surgery. 2019 May 15;43:1216-25. | Irrelevant |
| Flabouris A, Hart GK, George C. Outcomes of patients admitted to tertiary intensive care units after interhospital transfer: comparison with patients admitted from emergency departments. Crit Care Resusc. 2008 Jun 2;10(2):97-105. | Irrelevant |
| Fontebasso AM, Figueira S, Thavorn K, Glen P, Lampron J, Matar M. Financial implications of trauma patients at a Canadian level 1 trauma center: a retrospective cohort study. Trauma Surgery & Acute Care Open. 2020 Dec 1;5(1):e000568. | Irrelevant |
| Forristal C, Van Aarsen K, Columbus M, Wei J, Vogt K, Mal S. Predictors of hypothermia upon trauma center arrival in severe trauma patients transported to hospital via EMS. Prehospital emergency care. 2020 Jan 2;24(1):15-22. | Irrelevant |
| Fortuna GR, Mueller EW, James LE, Shutter LA, Butler KL. The impact of preinjury antiplatelet and anticoagulant pharmacotherapy on outcomes in elderly patients with hemorrhagic brain injury. Surgery. 2008 Oct 1;144(4):598-605. | Irrelevant |
| Fratianne RB, Brandt CP. Determining when care for burns is futile. The Journal of burn care & rehabilitation. 1997 May 1;18(3):262-7. | Irrelevant |
| Garwe T, Cowan LD, Neas B, Cathey T, Danford BC, Greenawalt P. Survival benefit of transfer to tertiary trauma centers for major trauma patients initially presenting to nontertiary trauma centers. Academic emergency medicine. 2010 Nov;17(11):1223-32. | Irrelevant |
| Garwe T, Cowan LD, Neas BR, Sacra JC, Albrecht RM. Directness of transport of major trauma patients to a level I trauma center: a propensity-adjusted survival analysis of the impact on short-term mortality. Journal of Trauma and Acute Care Surgery. 2011 May 1;70(5):1118-27. | Irrelevant |
| Giannoudis PV, Grotz MR, Tzioupis C, Dinopoulos H, Wells GE, Bouamra O, Lecky F. Prevalence of pelvic fractures, associated injuries, and mortality: the United Kingdom perspective. Journal of Trauma and Acute Care Surgery. 2007 Oct 1;63(4):875-83. | Irrelevant |
| Gioffrè-Florio M, Murabito LM, Visalli C, Pergolizzi FP, Famà F. Trauma in elderly patients: a study of prevalence, comorbidities and gender differences. Il Giornale di chirurgia. 2018 Jan;39(1):35. | Irrelevant |
| Gitajn IL, Castillo R, Breazeale S, Schoonover C, Berger P, Huang Y, O'Hara N, O'Toole RV, Sciadini MF. Survivorship after high-energy geriatric trauma. Journal of orthopaedic trauma. 2017 Aug 1;31(8):e230-5. | Irrelevant |
| Hackam DG, Kopp A, Redelmeier DA. Prognostic implications of warfarin cessation after major trauma: a population-based cohort analysis. Circulation. 2005 May 3;111(17):2250-6. | Irrelevant |
| Harcombe H, Davie G, Wyeth E, Ameratunga S, Powell D, Derrett S. Predictors of severe or multiple subsequent injuries over 24 months among an already-injured cohort in New Zealand. Injury. 2020 Mar 1;51(3):620-7. | Irrelevant |
| Harris I, Dao AT, Young J, Solomon M, Jalaludin BB, Rae H. Factors predicting patient satisfaction following major trauma. Injury. 2007 Sep 1;38(9):1102-8. | Irrelevant |
| Harris IA, Murgatroyd DF, Cameron ID, Young JM, Solomon MJ. The effect of compensation on health care utilisation in a trauma cohort. Medical Journal of Australia. 2009 Jun;190(11):619-22. | Irrelevant |
| Hasler RM, Nüesch E, Jüni P, Bouamra O, Exadaktylos AK, Lecky F. Systolic blood pressure below 110 mmHg is associated with increased mortality in penetrating major trauma patients: Multicentre cohort study. Resuscitation. 2012 Apr 1;83(4):476-81. | Irrelevant |
| Helm M, Hauke J, Kohler J, Lampl L. The concept of small volume resuscitation for preclinical trauma management: experiences in the Air Rescue Service. Der Unfallchirurg. 2013 Apr;116:326-31. | Irrelevant |
| Hibbard MR, Uysal S, Sliwinski M, Gordon W. Undiagnosed health issues in individuals with traumatic brain injury living in the community. The Journal of head trauma rehabilitation. 1998 Aug 1;13(4):47-57. | Irrelevant |
| Hoffman H, Furst T, Jalal MS, Chin LS. Costs and predictors of 30-day readmissions after craniotomy for traumatic brain injury: a nationwide analysis. Journal of neurosurgery. 2019 Aug 9;133(3):875-83. | Irrelevant |
| Hoffman H, Jalal MS, Chin LS. The risk factors, outcomes, and costs associated with venous thromboembolism after traumatic brain injury: a nationwide analysis. Brain Injury. 2019 Dec 6;33(13-14):1671-8. | Irrelevant |
| Holtslag HR, Post MW, van der Werken C, Lindeman E. Return to work after major trauma. Clinical Rehabilitation. 2007 Apr;21(4):373-83. | Irrelevant |
| Hsieh CH, Lin CC, Hsu SY, Hsieh HY. Pelvic fracture does not increase mortality in adult trauma patients: A propensity score analysis. Formosan Journal of Surgery. 2017 Nov 1;50(6):200-8. | Irrelevant |
| Hu J, Xia Q, Jiang Y, Zhou P, Li Y. Risk factors of indoor fall injuries in community-dwelling older women: a prospective cohort study. Archives of gerontology and geriatrics. 2015 Mar 1;60(2):259-64. | Irrelevant |
| Irgens I, Hoff JM, Jelnes R, Alexander M, Stanghelle JK, Thoresen M, Rekand T. Spinal cord injury and development of pressure injury during acute rehabilitation in Norway: a national retrospective cross-sectional study. Spinal Cord. 2020 Oct;58(10):1069-79. | Irrelevant |
| Justiniano CF, Evans DC, Cook CH, Eiferman DS, Gerlach AT, Beery II PR, Lindsey DE, Saum GE, Murphy CV, Miller SF, Papadimos TJ. Comorbidity-polypharmacy score: A novel adjunct in post–emergency department trauma triage. journal of surgical research. 2013 May 1;181(1):16-9. | Irrelevant |
| Katzenellenbogen JM, Atkins E, Thompson SC, Hersh D, Coffin J, Flicker L, Hayward C, Ciccone N, Woods D, Greenland ME, McAllister M. Missing voices: profile, extent, and 12-month outcomes of nonfatal traumatic brain injury in Aboriginal and non-Aboriginal adults in Western Australia using linked administrative records. The Journal of head trauma rehabilitation. 2018 Nov 1;33(6):412-23. | Irrelevant |
| Khorgami Z, Ewing KL, Mushtaq N, Chow GS, Howard CA. Predictors of discharge destination in patients with major traumatic injury: analysis of Oklahoma Trauma Registry. The American Journal of Surgery. 2019 Sep 1;218(3):496-500. | Irrelevant |
| Kim DY, Kobayashi L, Barmparas G, Fortlage D, Curry T, Coimbra R. Venous thromboembolism in the elderly: the result of comorbid conditions or a consequence of injury?. Journal of Trauma and Acute Care Surgery. 2012 May 1;72(5):1286-91. | Irrelevant |
| Kim J, Engelberg RA, Downey L, Lee RY, Powelson E, Sibley J, Lober WB, Curtis JR, Khandelwal N. Predictors of advance care planning documentation in patients with underlying chronic illness who died of traumatic injury. Journal of pain and symptom management. 2019 Nov 1;58(5):857-63. | Irrelevant |
| Kocuvan S, Brilej D, Stropnik D, Lefering R, Komadina R. Evaluation of major trauma in elderly patients–a single trauma center analysis. Wiener Klinische Wochenschrift. 2016 Dec;128:535-42. | Irrelevant |
| Kreutziger J, Schlaepfer J, Wenzel V, Constantinescu MA. The role of admission blood glucose in outcome prediction of surviving patients with multiple injuries. Journal of Trauma and Acute Care Surgery. 2009 Oct 1;67(4):704-8. | Irrelevant |
| Kubo J, Goldstein BA, Cantley LF, Tessier-Sherman B, Galusha D, Slade MD, Chu IM, Cullen MR. Contribution of health status and prevalent chronic disease to individual risk for workplace injury in the manufacturing environment. Occupational and environmental medicine. 2014 Mar 1;71(3):159-66. | Irrelevant |
| Kumar D, Bukhari H, Qureshi S. Three years mortality analysis in general surgery patients. Pakistan Journal of Medical Sciences. 2021 Jan;37(1):229. | Irrelevant |
| Lee HJ, Cheng CT, Chen CC, Liao CA, Chen SW, Wang SY, Wu YT, Hsieh CH, Yeh CN, Liao CH. Increased long-term pneumonia risk for the trauma-related splenectomized population-a population-based, propensity score matching study. Surgery. 2020 May 1;167(5):829-35. | Irrelevant |
| Lefering R, Paffrath T, Linker R, Bouillon B, Neugebauer EA, Deutsche Gesellschaft für Unfallchirurgie/German Society for Trauma Surgery. Head injury and outcome—what influence do concomitant injuries have?. Journal of Trauma and Acute Care Surgery. 2008 Nov 1;65(5):1036-44. | Irrelevant |
| Leitgeb J, Mauritz W, Brazinova A, Majdan M, Wilbacher I. Impact of concomitant injuries on outcomes after traumatic brain injury. Archives of orthopaedic and trauma surgery. 2013 May;133:659-68. | Irrelevant |
| Lien YC, Chen CH, Lin HC. Risk factors for 24-hour mortality after traumatic rib fractures owing to motor vehicle accidents: a nationwide population-based study. The Annals of thoracic surgery. 2009 Oct 1;88(4):1124-30. | Irrelevant |
| Lim S, Gangoli G, Adams E, Hyde R, Broder MS, Chang E, Reddy SR, Tarbox MH, Bentley T, Ovington L, Danker III W. Increased clinical and economic burden associated with peripheral intravenous catheter–related complications: Analysis of a US hospital discharge database. INQUIRY: The Journal of Health Care Organization, Provision, and Financing. 2019 Sep;56:0046958019875562. | Irrelevant |
| Little DM, Geary EK, Moynihan M, Alexander A, Pennington M, Glang P, Schulze ET, Dretsch M, Pacifico A, Davis ML, Stevens AB. Imaging chronic traumatic brain injury as a risk factor for neurodegeneration. Alzheimer's & Dementia. 2014 Jun;10:S188-95. | Irrelevant |
| Mai L, Spilsbury K, Edgar DW, Berghuber A, Wood FM. Increased risk of blood transfusion in patients with diabetes mellitus sustaining non-major burn injury. Burns. 2020 Jun 1;46(4):888-96. | Irrelevant |
| Manfredini R, Gallerani M, Boari B, Salmi R, Mehta RH. Seasonal variation in onset of pulmonary embolism is independent of patients' underlying risk comorbid conditions. Clinical and applied thrombosis/hemostasis. 2004 Jan;10(1):39-43. | Irrelevant |
| McChesney GC, Adamson G, Shevlin M. A latent class analysis of trauma based on a nationally representative sample of US adolescents. Social psychiatry and psychiatric epidemiology. 2015 Aug;50:1207-17. | Irrelevant |
| McGwin Jr G, George RL, Cross JM, Rue LW. Improving the ability to predict mortality among burn patients. Burns. 2008 May 1;34(3):320-7. | Irrelevant |
| McGwin G, May AK, Melton SM, Reiff DA, Rue LW. Recurrent trauma in elderly patients. Archives of Surgery. 2001 Feb 1;136(2):197-203. | Irrelevant |
| Metcalfe D, Olufajo O, Rios-Diaz AJ, Haider A, Havens JM, Nitzschke S, Cooper Z, Salim A. Are appendectomy outcomes in level I trauma centers as good as we think?. Journal of Surgical Research. 2016 May 15;202(2):239-45. | Irrelevant |
| Mitchell RJ, Cameron CM, McClure R. Quantifying the hospitalised morbidity and mortality attributable to traumatic injury using a population-based matched cohort in Australia. BMJ open. 2016 Dec 1;6(12):e013266. | Irrelevant |
| Mitchell RJ, Ting HP, Driscoll T, Braithwaite J. Identification and internal validation of models for predicting survival and ICU admission following a traumatic injury. Scandinavian journal of trauma, resuscitation and emergency medicine. 2018 Dec;26:1-1. | Irrelevant |
| Mizota T, Dong L, Takeda C, Shiraki A, Matsukawa S, Shimizu S, Kai S. Transient acute kidney injury after major abdominal surgery increases chronic kidney disease risk and 1-year mortality. Journal of critical care. 2019 Apr 1;50:17-22. | Irrelevant |
| Moscato BS, Trevisan M, Willer BS. The prevalence of traumatic brain injury and co-occurring disabilities in a national household survey of adults. The Journal of Neuropsychiatry and Clinical Neurosciences. 1994 Jan 1;6(2):134-42. | Irrelevant |
| Nakase-Richardson R, Schwartz DJ, Drasher-Phillips L, Ketchum JM, Calero K, Dahdah MN, Monden KR, Bell K, Magalang U, Hoffman JM, Whyte J. Comparative effectiveness of sleep apnea screening instruments during inpatient rehabilitation following moderate to severe TBI. Archives of physical medicine and rehabilitation. 2020 Feb 1;101(2):283-96. | Irrelevant |
| Nakase-Richardson R, Kamper JE, Garofano J, Schwartz DJ, Silva MA, Zeitzer J, Modarres M, Barnett SD. Concordance of actigraphy with polysomnography in traumatic brain injury neurorehabilitation admissions. Journal of Head Trauma Rehabilitation. 2016 Mar 1;31(2):117-25. | Irrelevant |
| Nan YY, Lu MS, Liu KS, Huang YK, Tsai FC, Chu JJ, Lin PJ. Blunt traumatic cardiac rupture: therapeutic options and outcomes. Injury. 2009 Sep 1;40(9):938-45. | Irrelevant |
| Nasr EM, Boroumand AB, KOLAHDOUZAN M. Early Intubation vs. Supportive CareOutcomes in Patients with Severe Chest Trauma; a randomized trial study. | Irrelevant |
| Nathens AB, Rivara FP, Wang J, Mackenzie EJ, Jurkovich GJ. Variation in the rates of do not resuscitate orders after major trauma and the impact of intensive care unit environment. Journal of Trauma and Acute Care Surgery. 2008 Jan 1;64(1):81-91. | Irrelevant |
| NeSmith EG, Weinrich SP, Andrews JO, Medeiros RS, Hawkins ML, Weinrich MC. Demographic differences in systemic inflammatory response syndrome score after trauma. American Journal of Critical Care. 2012 Jan 1;21(1):35-41. | Irrelevant |
| Nhac-Vu HT, Hours M, Charnay P, Chossegros L, Boisson D, Luaute J, Javouhey E, Ndiaye A, Laumon B. Predicting self-reported recovery one year after major road traffic accident trauma. Journal of rehabilitation medicine. 2011 Jan 1;43(9):pp-776. | Irrelevant |
| Nishimura T, Naito H, Fujisaki N, Ishihara S, Nakao A, Nakayama S. The psoas muscle index as a predictor of mortality and morbidity of geriatric trauma patients: experience of a major trauma center in Kobe. Surgery today. 2020 Sep;50:1016-23. | Irrelevant |
| Okazaki T, Hifumi T, Kawakita K, Nakashima R, Matsumoto A, Shishido H, Ogawa D, Okauchi M, Shindo A, Kawanishi M, Tamiya T. Association between comorbidities, nutritional status, and anticlotting drugs and neurologic outcomes in geriatric patients with traumatic brain injury. World neurosurgery. 2016 Sep 1;93:336-40. | Irrelevant |
| Passias PG, Poorman GW, Segreto FA, Jalai CM, Horn SR, Bortz CA, Vasquez-Montes D, Diebo BG, Vira S, Bono OJ, De La Garza-Ramos R. Traumatic fractures of the cervical spine: analysis of changes in incidence, cause, concurrent injuries, and complications among 488,262 patients from 2005 to 2013. World neurosurgery. 2018 Feb 1;110:e427-37. | Irrelevant |
| Patel P, Taylor D, Park MS. Characteristics of traumatic brain injury during Operation Enduring Freedom–Afghanistan: a retrospective case series. Neurosurgical focus. 2019 Nov 1;47(5):E13. | Irrelevant |
| Peck KA, Calvo RY, Sise CB, Johnson J, Yen JW, Sise MJ, Dunne CE, Badiee J, Shackford SR, Lobatz MA. Death after discharge: predictors of mortality in older brain-injured patients. Journal of trauma and acute care surgery. 2014 Dec 1;77(6):978-83. | Irrelevant |
| Phillips B, Turco L, McDonald D, Mause A, Walters RW. Penetrating injuries to the duodenum: An analysis of 879 patients from the National Trauma Data Bank, 2010 to 2014. Journal of trauma and acute care surgery. 2017 Nov 1;83(5):810-7. | Irrelevant |
| Pugh MJ, Swan AA, Carlson KF, Jaramillo CA, Eapen BC, Dillahunt-Aspillaga C, Amuan ME, Delgado RE, McConnell K, Finley EP, Grafman JH. Traumatic brain injury severity, comorbidity, social support, family functioning, and community reintegration among veterans of the Afghanistan and Iraq wars. Archives of physical medicine and rehabilitation. 2018 Feb 1;99(2):S40-9. | Irrelevant |
| Rao V, Spiro J, Vaishnavi S, Rastogi P, Mielke M, Noll K, Cornwell E, Schretlen D, Makley M. Prevalence and types of sleep disturbances acutely after traumatic brain injury. Brain injury. 2008 Jan 1;22(5):381-6. | Irrelevant |
| Rau CS, Wu SC, Hsu SY, Liu HT, Huang CY, Hsieh TM, Chou SE, Su WT, Liu YW, Hsieh CH. Concurrent types of intracranial hemorrhage are associated with a higher mortality rate in adult patients with traumatic subarachnoid hemorrhage: a cross-sectional retrospective study. International Journal of Environmental Research and Public Health. 2019 Dec;16(23):4787. | Irrelevant |
| Rau CS, Wu SC, Kuo PJ, Chen YC, Chien PC, Hsieh HY, Hsieh CH. Same abbreviated injury scale values may be associated with different risks to mortality in trauma patients: a cross-sectional retrospective study based on the trauma registry system in a level I trauma center. International journal of environmental research and public health. 2017 Dec;14(12):1552. | Irrelevant |
| Redelmeier DA, Naylor CD, Brenneman FD, Sharkey PW, Juurlink DN. Major trauma in elderly adults receiving lipid-lowering medications. Journal of Trauma and Acute Care Surgery. 2001 Apr 1;50(4):678-83. | Irrelevant |
| Renfro M, Bainbridge DB, Smith ML. Validation of evidence-based fall prevention programs for adults with intellectual and/or developmental disorders: a modified Otago exercise program. Frontiers in public health. 2016 Dec 6;4:261. | Irrelevant |
| Richmond TS, Thompson HJ, Kauder D, Robinson KM, Strumpf NE. A feasibility study of methodological issues and short-term outcomes in seriously injured older adults. American Journal of Critical Care. 2006 Mar 1;15(2):158-65. | Irrelevant |
| Ryan ML, Ogilvie MP, Pereira BM, Gomez-Rodriguez JC, Manning RJ, Vargas PA, Duncan RC, Proctor KG. Heart rate variability is an independent predictor of morbidity and mortality in hemodynamically stable trauma patients. Journal of Trauma and Acute Care Surgery. 2011 Jun 1;70(6):1371-80. | Irrelevant |
| Sabaz M, Simpson GK, Walker AJ, Rogers JM, Gillis I, Strettles B. Prevalence, comorbidities, and correlates of challenging behavior among community-dwelling adults with severe traumatic brain injury: a multicenter study. The Journal of Head Trauma Rehabilitation. 2014 Mar 1;29(2):E19-30. | Irrelevant |
| Saleh M, Saatchi R, Burke D. Analysis of the influence of trauma injury factors on the probability of survival. International journal of biology and biomedical engineering. 2017 Nov 24;11:88-96. | Irrelevant |
| Samuel BS. The influence of insurance status on the surgical treatment of acute spinal fractures. Spine. 2016 Jan;41(1):E37. | Irrelevant |
| Samuelsson C, Sjöberg F, Karlström G, Nolin T, Walther SM. Gender differences in outcome and use of resources do exist in Swedish intensive care, but to no advantage for women of premenopausal age. Critical Care. 2015 Dec;19(1):1-9. | Irrelevant |
| Schneider EB, Efron DT, MacKenzie EJ, Rivara FP, Nathens AB, Jurkovich GJ. Premorbid statin use is associated with improved survival and functional outcomes in older head-injured individuals. Journal of Trauma and Acute Care Surgery. 2011 Oct 1;71(4):815-9. | Irrelevant |
| Shamim MS, Qadeer M, Murtaza G, Enam SA, Farooqi NB. Emergency department predictors of tracheostomy in patients with isolated traumatic brain injury requiring emergency cranial decompression. Journal of neurosurgery. 2011 Nov 1;115(5):1007-12. | Irrelevant |
| Sharwood LN, Whyatt D, Vaikuntam BP, Cheng CL, Noonan VK, Joseph AP, Ball J, Stanford RE, Kok MR, Withers SR, Middleton JW. A geospatial examination of specialist care accessibility and impact on health outcomes for patients with acute traumatic spinal cord injury in New South Wales, Australia: a population record linkage study. BMC Health Services Research. 2021 Dec;21(1):1-3. | Irrelevant |
| Shi HY, Hwang SL, Lee IC, Chen IT, Lee KT, Lin CL. Trends and outcome predictors after traumatic brain injury surgery: a nationwide population-based study in Taiwan. Journal of neurosurgery. 2014 Dec 1;121(6):1323-30. | Irrelevant |
| Shinoda J, Nagamine Y, Kobayashi S, Odaki M, Oka N, Kinugasa K, Nakamura H, Ichida T, Miyashita R, Shima H, Hama T. Multidisciplinary attentive treatment for patients with chronic disorders of consciousness following severe traumatic brain injury in the NASVA of Japan. Brain Injury. 2019 Dec 6;33(13-14):1660-70. | Irrelevant |
| Skaansar O, Tverdal C, Rønning PA, Skogen K, Brommeland T, Røise O, Aarhus M, Andelic N, Helseth E. Traumatic brain injury—the effects of patient age on treatment intensity and mortality. BMC neurology. 2020 Dec;20(1):1-0. | Irrelevant |
| Stein TD, Crary JF. Chronic traumatic encephalopathy and neuropathological comorbidities. InSeminars in neurology 2020 Jun 30 (Vol. 40, No. 04, pp. 384-393). 333 Seventh Avenue, New York, NY 10001, USA.: Thieme Medical Publishers. | Irrelevant |
| Tran DD, Cuesta MA, Oe PL. Acute renal failure in patients with severe civilian trauma. Nephrology, Dialysis, Transplantation: Official Publication of the European Dialysis and Transplant Association-European Renal Association. 1994 Jan 1;9:121-5. | Irrelevant |
| Tran DD, Cuesta MA, Van Leeuwen PA, Nauta JJ, Wesdorp RI. Risk factors for multiple organ system failure and death in critically injured patients. Surgery. 1993 Jul 1;114(1):21-30. | Irrelevant |
| Trivedi N, Ylagan M, Moore TR, Bansal V, Wolfson T, Fortlage D, Coimbra R, Kelly T. Predicting adverse outcomes following trauma in pregnancy. The Journal of Reproductive Medicine. 2012 Jan 1;57(1-2):3-8. | Irrelevant |
| Vallier HA, Wang X, Moore TA, Wilber JH, Como JJ. Timing of orthopaedic surgery in multiple trauma patients: development of a protocol for early appropriate care. Journal of orthopaedic trauma. 2013 Oct 1;27(10):543-51. | Irrelevant |
| Vanzant EL, Lopez CM, Ozrazgat-Baslanti T, Ungaro R, Davis R, Cuenca AG, Gentile LF, Nacionales DC, Cuenca AL, Bihorac A, Leeuwenburgh C. Persistent inflammation, immunosuppression and catabolism syndrome after severe blunt trauma. The journal of trauma and acute care surgery. 2014 Jan;76(1):21. | Irrelevant |
| Verma A, Anand V, Verma NP. Sleep disorders in chronic traumatic brain injury. Journal of Clinical Sleep Medicine. 2007 Jun 15;3(4):357-62. | Irrelevant |
| Wang HE, Balasubramani GK, Cook LJ, Yealy DM, Lave JR. Medical conditions associated with out-of-hospital endotracheal intubation. Prehospital Emergency Care. 2011 Jun 8;15(3):338-46. | Irrelevant |
| Williams H, Chitsabesan P, Lennox C, Tariq O, Shaw J. Traumatic brain injury in juvenile offenders: findings from the comprehensive health assessment tool study and the development of a specialist linkworker service. Journal of head trauma rehabilitation. 2015 Mar 1;30(2):106-15. | Irrelevant |
| Winfield RD, Delano MJ, Dixon DJ, Schierding WS, Cendan JC, Lottenberg L, Lopez MC, Baker HV, Cobb JP, Moldawer LL, Maier RV. Differences in outcome between obese and nonobese patients following severe blunt trauma are not consistent with an early inflammatory genomic response. Critical care medicine. 2010 Jan;38(1):51. | Irrelevant |
